# A Review of Infectious and Non‐Infectious Causes of Pregnancy Loss in Goats

**DOI:** 10.1111/rda.70198

**Published:** 2026-03-24

**Authors:** Friederike Maria Kaus, Hannah Eggimann, Barbara Tschulena, Gaby Hirsbrunner, Patrik Zanolari

**Affiliations:** ^1^ Clinic for Ruminants, Department of Clinical Veterinary Science, Vetsuisse‐Faculty University of Bern Bern Switzerland; ^2^ Nutztierpraxis Rudolph AG Hochdorf LU Switzerland; ^3^ Praxis Dr. Hutter Zuzwil BE Switzerland; ^4^ Clinical Centre for Ruminant and Camelid Medicine, Clinical Department for Farm Animals and Food System Science University of Veterinary Medicine Vienna Vienna Austria

**Keywords:** abortion, goat, neonatal death, perinatal mortality, pregnancy loss, stillbirth

## Abstract

Goats play a vital role in global agriculture, particularly in developing regions, and are increasingly kept as companion animals. Given their economic and social importance, pregnancy losses in goats represent a substantial challenge, leading to considerable economic losses and raising concerns about animal welfare and public health, as some etiological agents are zoonotic. Understanding both infectious and non‐infectious causes of pregnancy loss in goats is therefore essential for effective prevention and management. However, to date, no scoping review has systematically mapped the existing literature or identified current knowledge gaps. This article aims to provide a comprehensive overview of the available evidence on the causes of pregnancy loss in goats and to propose directions for future research.

## 1 Introduction

The global goat population has more than tripled between 1961 and 2023, now standing at approximately 1.2 billion animals (FAOSTAT Database 2025). As highly adaptable animals capable of thriving under extreme conditions (Alexandre and Mandonnet 2005), goats are the most widespread domesticated species worldwide (Alexandre and Mandonnet 2005). They are particularly raised for meat, milk, and fibre production, making them economically important, especially in developing regions across Asia and Africa (Boyazoglu et al. 2005; Dubey et al. 2020; Rakib et al. 2022). In addition, goats are increasingly kept as companion animals, providing various benefits (Friedman and Krause‐Parello 2018). Reproductive performance is one of the most critical factors influencing economic efficiency in goat production (Quan et al. 2025), with pregnancy loss representing a major constraint (Ali et al. 2022). Such losses affect not only individual animals but may also contribute to the spread of infection among herds, other species, and even humans, as many causative pathogens are zoonotic. In addition to infectious agents, non‐infectious factors can also lead to pregnancy loss. Understanding the aetiology, diagnosis, transmission, and prevention of abortion and stillbirth is therefore essential to mitigate their negative impacts. Despite their global relevance, goats are often considered a minor species in research, and substantial knowledge gaps remain. Moreover, no scoping review has yet systematically mapped the existing literature on this topic. Therefore, the present article aims to clarify the current state of knowledge, summarise previous research, and identify existing knowledge gaps to support future research directions.

## 2 Material and Methods

**FIGURE 1:**
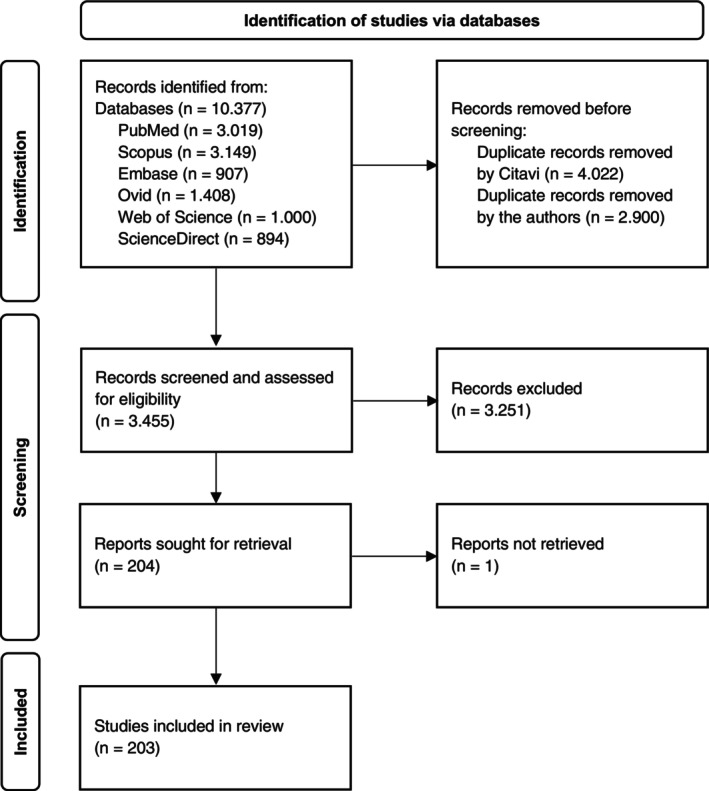
PRISMA flow diagram of the selection process.

To provide an overview of the currently available data on pregnancy loss in goats, this scoping review was conducted in accordance with the Joanna Briggs Institute (JBI) methodology (Peters et al. 2024) and reported following the PRISMA‐ScR guidelines (Tricco et al. 2018). The review process was based on the framework developed by Arksey and O'Malley (2005), later refined by the JBI (Peters et al. 2024). It was adapted and structured into six steps: (1) defining and aligning the objective and question, (2) developing and aligning the inclusion criteria with the objective and question, (3) searching for the evidence, (4) selecting the evidence, (5) extracting the evidence, and (6) presentation of the results (Peters et al. 2024). Regarding the first step, the primary question guiding this review was: ‘What publications exist on the infectious and non‐infectious causes of pregnancy loss in goats?’ Appropriate search terms were identified, including: goat, abortion, pregnancy loss, embryonic death, stillbirth, pathologic pregnancy, neonatal loss, perinatal mortality, neonatal death; Border disease, Schmallenberg, bluetongue; chlamydiosis, coxiellosis, brucellosis, campylobacteriosis, leptospirosis, listeriosis, salmonellosis; neosporosis, toxoplasmosis; trace minerals. Inclusion criteria were defined and applied in the subsequent search. Searches were conducted in spring 2025 across six databases: PubMed, Scopus, Embase, Ovid, Web of Science and ScienceDirect. Database‐specific filters were applied to optimise the relevance, and searches included titles, abstracts, and keywords. Publications not written in English, German, or French as well as those published before 1990 were excluded. Duplicates were removed using Citavi (Lumivero, Denver, USA) and manually by the authors. In the fourth step, titles, abstracts, and, when necessary, full texts of the remaining publications were screened and listed in Microsoft Excel (Microsoft Corporation, Redmond, USA). Exclusion criteria included studies not focusing on goats, non‐peer‐reviewed articles, reports of embryonal losses up to 42 days of gestation or deaths occurring more than 48 h after birth, publications exclusively addressing billy goats, investigations limited to the dam, experimental infections, and studies limited to molecular‐level findings. The remaining articles underwent full‐text review to determine final eligibility (Figure 1). In the fifth step, relevant data were systematically extracted, and finally, in the sixth step, the data were compiled, clearly organised and presented in a Microsoft Word table (Microsoft Corporation, Redmond, USA).

## 3 Results

### 3.1 Search Results

As part of the identification process, the search across the six databases using the inclusion criteria specified under ‘Material and methods’ yielded 10,377 records. These publications were imported into a combined project in the reference management software Citavi. During this process, 4022 duplicates were automatically detected and removed. An additional 2900 duplicates were manually excluded, leaving 3455 publications undergoing screening. Titles and abstracts were reviewed, and, where necessary, full‐text articles were assessed for eligibility. Through this procedure, non‐relevant records were excluded. One publication was not available, and 203 publications were ultimately included in the review. The selection process is presented (Figure 1).

**FIGURE 2:**
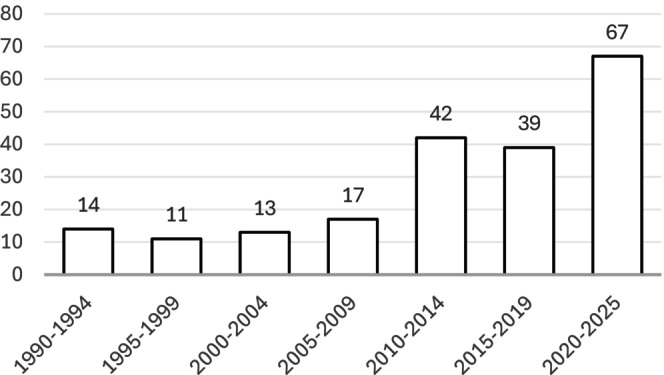
Number of included studies by 5‐year interval (1990–2025).

Most relevant studies (*n* = 67) were published between 2020 and early 2025, whereas only 14 suitable publications were identified for the period 1990–1994 and 11 for 1995–1999, as illustrated by the number of publications per period (Figure 2).

**FIGURE 3:**
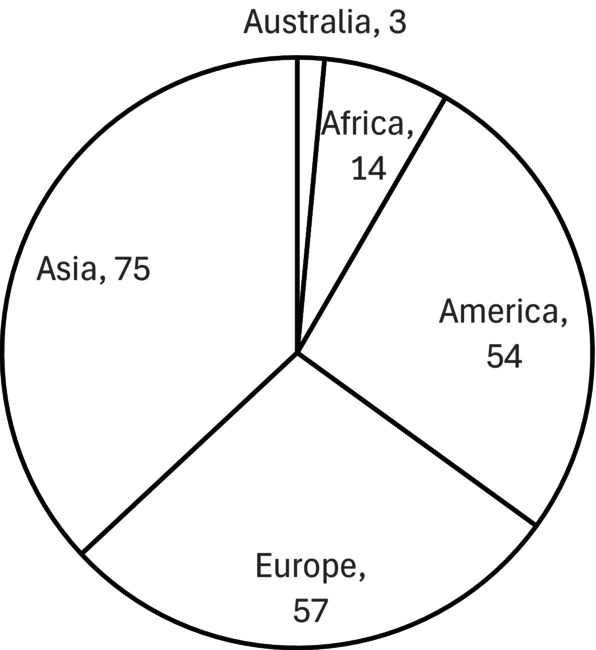
Geographical origin of included studies by continent.

The majority of studies included in this review were conducted in Asia (*n* = 75), followed by Europe (*n* = 57), and America (*n* = 54), while only 14 and three publications originated from Africa and Australia, respectively (Figure 3).

The most extensively studied abortifacients in goats are *Coxiella burnetii* (*n* = 40), *Toxoplasma gondii* (*n* = 33), and *Chlamydia abortus* (*n* = 26).

A summary of the search results on infectious and non‐infectious causes of pregnancy loss in goats is provided in the Appendix S1.

### 3.2 Infectious Agents

Reported detection rates of infectious agents in caprine abortion cases were 24.5% (Esmaeili et al. 2025), 25% (19 of 75) (Szeredi et al. 2006) and 37% (Moeller Jr 2001).

#### 3.2.1 Viral Pathogens

1.5% (Esmaeili et al. 2025) to 2% (Moeller Jr 2001) of abortions in goats have been associated with viral pathogens.

##### 3.2.1.1 Flaviviridae

###### 3.2.1.1.1 Pestiviruses

Evidence of pestiviruses has been reported in studies from Turkey (Albayrak and Özan 2012; Sakmanoğlu et al. 2021; Şevik 2021; Tuncer‐Göktuna et al. 2016), Israel (Golender et al. 2021) and the Iberian Peninsula (Alzuguren et al. 2023). Interspecies transmission between cattle and goats is considered possible (Broaddus et al. 2009; Tuncer‐Göktuna et al. 2016).

###### 3.2.1.1.2 Border Disease Virus (BDV)

Border disease pestivirus was detected in 0.8% (Esmaeili et al. 2025) and 1.2% of caprine pregnancy losses (Ramo et al. 2022) and associated with late‐term abortions, neonatal deaths, and the birth of weak or small kids (Rosamilia et al. 2014; Sharawi et al. 2010; Toplu et al. 2011). Some affected herds exhibited only increased abortion rates (Sharawi et al. 2010), while some kids showed neurological signs, such as ataxia, paresis, paralysis, and tremors (Toplu et al. 2011). Gross lesions included deformities of the limbs, internal organs and head, along with central nervous system abnormalities such as hydrocephaly, porencephaly and hypoplasia (Sharawi et al. 2010; Toplu et al. 2011). Subcutaneous edema and ascites were also described (Rosamilia et al. 2014). Histopathology revealed nonsuppurative necrotizing meningoencephalomyelitis, accompanied by multifocal white matter lesions with hypomyelinogenesis and demyelination, gliosis, perivascular cuffing, malacia and mineralisation, as well as dilated meningeal vessels (Sharawi et al. 2010; Toplu et al. 2011). Hepatic necrobiosis and vacuolar degeneration were also observed (Sharawi et al. 2010).

###### 3.2.1.1.3 Bovine Viral Diarrhoea Virus (BVDV)

After commingling with persistently infected heifers, 12 of 24 pregnant goats aborted due to bovine viral diarrhoea virus (BVDV) type 2a infection. Affected does showed placentitis, and foetuses exhibited facial deformities. Histological examination identified necrotizing placentitis, foetal myocarditis, thymic atrophy, and inflammation of the choroid plexus and encephalon (Broaddus et al. 2009). BVDV antigen was identified in placental, cardiac, thymic and brain tissues (Lamm et al. 2009).

###### 3.2.1.1.4 HoBi‐Like Pestivirus

HoBi‐like pestivirus (BVDV‐3), a genetically distinct but BVDV‐related pestivirus, is suspected to contribute to pregnancy loss in goats and was detected in third‐trimester aborted foetuses in China (Shi et al. 2023).

##### 3.2.1.2 Herpesviridae

###### 3.2.1.2.1 Caprine Herpesvirus (CpHV)

Evidence of caprine herpesvirus (CpHV) has been identified in 2% of aborted foetuses (Moeller Jr 2001). Pregnancy loss can occur at various gestational stages (Gonzalez et al. 2017; Williams et al. 1997), with late‐gestation abortion storms also reported (McCoy et al. 2007; Uzal et al. 2004). Does typically show no clinical signs aside from abortion and stillbirth (Chénier et al. 2004; Uzal et al. 2004; Williams et al. 1997), and in twin pregnancies, usually one kid is born alive while the other is stillborn (Chénier et al. 2004; Gonzalez et al. 2017). Aborted foetuses are often autolyzed (Chénier et al. 2004; Moeller Jr 2001; Uzal et al. 2004; Williams et al. 1997), although some present multifocal pinpoint white foci with hyperemia in the lungs and liver (Chénier et al. 2004; Uzal et al. 2004), while other studies report no gross lesions (Gonzalez et al. 2017; Williams et al. 1997). Histologically, multifocal coagulative necrosis of the liver, lungs, spleen, adrenal glands, kidneys and brain with intranuclear eosinophilic inclusion bodies has been described (Chénier et al. 2004; Gonzalez et al. 2017; McCoy et al. 2007; Moeller Jr 2001; Uzal et al. 2004; Williams et al. 1997).

##### 3.2.1.3 Paramyxoviridae

###### 3.2.1.3.1 Peste Des Petits Ruminants Virus (PPRV)

Evidence of peste des petits ruminants virus (PPRV) lineage IV was confirmed in 4.1% of 74 (Murat 2024) and 11.5% of 26 caprine aborted foetuses, and was not related to vaccine strains (Pestil et al. 2020). Affected does exhibited mild respiratory and digestive signs, without severe disease or fatalities (Murat 2024; Pestil et al. 2020). Abortions occurred at all stages of pregnancy, more frequently during the first 2 months, possibly due to immature foetal immunity. Coinfections were ruled out, and viral presence was confirmed in both dam and aborted foetus, indicating that other pestiviruses are not required as co‐factors and that abortion does not occur solely due to oxidative stress. Foetuses showed no gross abnormalities, except for congested and edematous lungs (Murat 2024).

##### 3.2.1.4 Peribunyaviridae

###### 3.2.1.4.1 Simbu Serogroup

Akabane virus (AKAV), a vector‐borne pathogen, was detected in an aborted goat foetus in Turkey. No gross abnormalities such as arthrogryposis‐hydranencephaly syndrome (AHS) were observed. Infections tend to re‐emerge periodically as herd immunity wanes following endemic periods (Cagirgan et al. 2022). AKAV was also detected in placental and foetal tissues of abortion cases in Israel (Golender et al. 2021).

Evidence of Schmallenberg virus (SBV), which emerged in autumn 2011 and has been associated with pregnancy loss in goats, has been reported in studies from France (Dominguez et al. 2014) and Germany (Herder et al. 2012; Wagner et al. 2014). Aborted foetuses typically exhibited AHS, accompanied by skull asymmetry, brachygnathia inferior, pulmonary hypoplasia, and cerebellar hypoplasia or aplasia (Dominguez et al. 2014; Herder et al. 2012; Wagner et al. 2014). Microscopic examination revealed perivascular lymphohistiocytic poliomyelitis, astrogliosis, and microgliosis (Herder et al. 2012). As an arthropod‐borne virus, SBV infection shows a seasonal pattern, with abortions resulting from infections acquired during the previous vector season and a minimal interval of approximately 3 months between infection and birth (Dominguez et al. 2014).

Shamonda virus (SHAV) was detected in an aborted goat foetus in South Africa, which showed no gross lesions. Meconium aspiration, likely resulting from foetal distress, and mononuclear perivascular cuffing due to virus‐induced meningoencephalitis were observed. The arboviral genome was identified and found to be similar to sequences detected in *Culicoides* midges from northern South Africa (van der Walt et al. 2023).

An aborted kid showing hydrocephalus was found to be infected with the vector‐borne Shuni virus (SHUV), which was linked to late‐term abortion in further does (Golender et al. 2022). Additional cases of SHUV have been reported in Israel (Golender et al. 2021).

###### 3.2.1.4.2 Bunyamwera Serogroup

Cache Valley virus (CVV), an arbovirus, was identified as the most likely abortifacient in a goat that delivered a weak kid and a stillborn, malformed foetus shortly before term. Gross lesions included AHS, accompanied by reduced muscle mass, scoliosis and torticollis. Histopathological examination revealed hydranencephaly, neuronal loss in the brainstem, and skeletal muscle hypoplasia (Edwards et al. 2003).

##### 3.2.1.5 Sedoreoviridae

###### 3.2.1.5.1 Bluetongue Virus (BTV)

In an Indian herd, 52% of pregnant goats experienced abortions during the third month of gestation. Affected does showed weight loss, and bluetongue virus serotype 1 was isolated from the foetuses, indicating transplacental transmission. As an arbovirus, BTV‐1 was also detected in *Culicoides* vectors collected at the farm (Chauhan et al. 2014). In Israel, serotype 4 was associated with caprine abortions in 2018 (Golender et al. 2020), and RT‐PCR for pan‐BTV was positive in an aborted goat foetus in 2019 (Golender et al. 2021).

BTV vaccine strains from an aborted doe vaccinated with a modified live vaccine were identified in a foetal spleen, demonstrating transplacental infection leading to abortion (Savini et al. 2014).

#### 3.2.2 Bacterial Pathogens

Abortions linked to bacterial agents accounted for 30.5% (Moeller Jr 2001) to 35.8% (Esmaeili et al. 2025) of cases.

##### 3.2.2.1 Brucella

*Brucella* spp. are well‐established infectious agents associated with pregnancy loss in goats (Maksimović et al. 2022).

Evidence of *Brucella melitensis* ( *B. melitensis* ) in goat foetuses is described as the most common infectious abortifacient in goat herds in Iran (Esmaeili et al. 2025). Most publications refer to biovars 1, 2 and 3; however, *B. melitensis* biovar 9 has also been identified in one late‐gestation aborted foetus (Al‐Majali 2005). *B. melitensis* biovar 1 was detected in a foetus from a herd in which five out of seven pregnant does aborted (Ribeiro et al. 1990) and has also been reported in other publications originating from Africa and Asia (Abnaroodheleh et al. 2021; Aldomy et al. 1992; Behroozikhah et al. 2022; Büyükcangaz et al. 2009; Dadar and Alamian 2021a; Dadar et al. 2022; Demirpence et al. 2022). Biovar 2 has been identified in aborted foetuses in Iran (Dadar and Alamian 2021a, 2021b; Dadar et al. 2023). Biovar 3 has been associated with late‐term abortions and preterm delivery (Al‐Majali 2005), and affected foetuses showed edema as well as leukocytic bronchopneumonia (Al‐Ani et al. 2004). Evidence of biovar 3 in foetal membranes and stomach contents has been further demonstrated in studies from Asia (Demirpence et al. 2022; Singh et al. 2013). Detection rates of *B. melitensis* DNA in abortion material were 7.8% in a study of 51 samples (Sanjay Ghodasara et al. 2010) and 16.4% in 140 samples (Moshkelani et al. 2011), and reported incidences of *B. melitensis* ‐related abortions ranged from 18% (Atwa and Rady 2007) to 27% (Al‐Majali 2005).

A mixed infection of *B. melitensis* and *B. abortus* was identified in an abortion storm in Rwanda, affecting 24 of 40 does (Ntivuguruzwa et al. 2022). In addition, *B. ovis* was detected in an aborted goat foetus in Iran (Alirezaei et al. 2024).

##### 3.2.2.2 Campylobacter

The reported prevalence of *Campylobacter*‐associated abortions ranges from 0.9% (Moeller Jr 2001) to 3.9% (Ramo et al. 2022). Autolysis and purulent placentitis were described in *Campylobacter‐*positive foetuses from the Netherlands, without additional abnormalities (van den Brom et al. 2012). Coinfections were reported in 2.4% of cases (Ramo et al. 2022) and are presented in the corresponding chapter.

*Campylobacter fetus* ( *C. fetus* ) was identified in samples from the Eastern Mediterranean region (Atwa and Rady 2007; Omidi 2015; Sakmanoğlu et al. 2021). This species is associated with abortions occurring during the last trimester, while affected does showed no other clinical symptoms (Sakmanoğlu et al. 2021). Specifically, both *C. fetus* ssp. *fetus* and *C. fetus* ssp. *venerealis* have been detected (Atwa and Rady 2007).

*Campylobacter jejuni* has been found in the USA (Moeller Jr 2001; Sahin et al. 2012), Brazil (Scarcelli et al. 2009), and South Africa (Jonker et al. 2023). *Campylobacter jejuni* ssp. *jejuni* was also identified in third‐trimester aborted foetuses exhibiting suppurative pneumonia and multifocal hepatic necrosis (Moeller Jr 2001), as well as meningoencephalitis (Jonker et al. 2023).

##### 3.2.2.3 Chlamydia

*Chlamydia* spp. are among the most frequently identified bacterial pathogens in aborted caprine tissues (Esmaeili et al. 2025; Szeredi et al. 2020; van Engelen et al. 2014), typically associated with necrosuppurative placentitis and multifocal necrosuppurative hepatitis and pneumonia in aborted foetuses (Szeredi et al. 2020).

*Chlamydia abortus* ( *C. abortus* ) was the most commonly detected abortifacient, with detection rates ranging from 14.6% (Şevik 2024) up to 37% (Hazlett et al. 2013) in Switzerland (Chanton‐Greutmann et al. 2002), Hungary (Szeredi et al. 2006), Canada (Hazlett et al. 2013), Turkey (Şevik 2024), and in the Netherlands in 2015–2016 (van den Brom et al. 2021). It was also the second most frequent cause on the Iberian Peninsula (Alzuguren et al. 2023; Ramo et al. 2022), in Iran (Esmaeili et al. 2025), and during the lambing seasons between 2006 and 2011 in the Netherlands (van den Brom et al. 2012). In contrast, *C. abortus* does not play a significant role in pregnancy loss in Sardinia (Masala et al. 2005). Most affected does aborted in the last trimester (Chanton‐Greutmann et al. 2002; Şevik 2024), showing no clinical symptoms (Heidari et al. 2018; Şevik 2024). Pathological findings typically revealed necropurulent placentitis and vasculitis, and multifocal necropurulent hepatitis in the foetuses (Chanton‐Greutmann et al. 2002; Di Paolo et al. 2019; Hailat et al. 2018; Islam et al. 2022; Kalender et al. 2013; Szeredi et al. 2006; van den Brom et al. 2012), although some cases lacked gross or histological abnormalities (Jonker et al. 2023; van den Brom et al. 2021). Reported risk factors for *C. abortus* ‐related abortion include inadequate hygiene, animal movement, introduction of new animals, and inter‐animal contact (Chanton‐Greutmann et al. 2002; Fayez et al. 2021; Twomey et al. 2011). Abortions occur more frequently in primiparous does (Chanton‐Greutmann et al. 2002; Heidari et al. 2018), and typically display an enzootic pattern, with abortion rates decreasing below 5% in subsequent kidding seasons (Chanton‐Greutmann et al. 2002). Coinfections with *C. burnetii* have been detected (Hazlett et al. 2013; Kreizinger et al. 2015; Ramo et al. 2022; Şevik 2024).

In the USA, *Chlamydia psittaci* has been identified as the most common cause of pregnancy loss (Moeller Jr 2001). Positive samples derived from last‐trimester aborted foetuses showing no (Escalante‐Ochoa et al. 1997) to mild gross lesions, such as swollen hemorrhagic liver (Liao et al. 1997), necrotic hepatitis, splenitis, as well as placentitis and vasculitis (Moeller Jr 2001). Affected does remained asymptomatic (Escalante‐Ochoa et al. 1997).

*Chlamydia pecorum* is described as an abortifacient in one twin abortion at approximately 130 days of gestation. Macroscopic lesions included anasarca and intermuscular edema, accompanied by brachygnathia and palatoschisis. Placental cotyledons were coated with a fibrinopurulent exudate, and histology confirmed necrosuppurative placentitis with arteriolitis and thrombosis, as well as foetal hepatitis and fibrinopurulent enteritis (Giannitti et al. 2016).

##### 3.2.2.4 *Coxiella burnetii*

Evidence of *C. burnetii* in caprine abortion material is among the most frequently reported findings (Alzuguren et al. 2023; Chanton‐Greutmann et al. 2002; Esmaeili et al. 2025; Moeller Jr 2001; Navarro et al. 2009; Ramo et al. 2022; Şevik 2024; van den Brom et al. 2012). Detection rates range from 2.9% to 11% (Chanton‐Greutmann et al. 2002; Esmaeili et al. 2025; Moeller Jr 2001; van den Brom et al. 2012) until 43% (Ramo et al. 2022).

Abortions typically occur during late‐term pregnancy (Copeland et al. 1991; Moeller Jr 2001; Reichel et al. 2012; Şevik 2024; Twomey et al. 2011). Gross lesions are often absent in foetuses, while placentae exhibit characteristic thickened, leathery intercotyledonary areas (Copeland et al. 1991; Moore et al. 1991; Rajagunalan et al. 2019; Sanford et al. 1994; Szeredi et al. 2006; van den Brom et al. 2012). Microscopically, lesions include necrosuppurative intercotyledonary placentitis, vasculitis, and degenerated chorionic cells (Álvarez‐Alonso et al. 2018; Moeller Jr 2001; Moore et al. 1991; Sanford et al. 1993, 1994; Szeredi et al. 2006; Twomey et al. 2011; van den Brom et al. 2012). Occasionally, foetal granulomatous hepatitis or interstitial nonsuppurative pneumonia has been described (Moeller Jr 2001; Moore et al. 1991; van den Brom et al. 2012). *C. burnetii* can be shed in vaginal mucus for up to 5 months post‐abortion (Álvarez‐Alonso et al. 2018; Heinzelmann et al. 2020), leading to substantial environmental contamination (Álvarez‐Alonso et al. 2018; Reichel et al. 2012). Outbreaks have been linked to animal movements (Sanford et al. 1993, 1994), and coinfections, mainly with *Chlamydia abortus*, are reported (Ramo et al. 2022; Şevik 2024).

##### 3.2.2.5 *Escherichia coli*

*Escherichia coli* ( *E. coli* ) was detected in 1.3%–7.1% of caprine abortion cases (Esmaeili et al. 2025; Moeller Jr 2001; Omidi 2015; Szeredi et al. 2006). Most *E. coli* ‐associated abortions occurred during the last trimester (Moeller Jr 2001). Foetuses often showed no gross lesions (Szeredi et al. 2006), whereas histopathological findings commonly included purulent pneumonia, peritonitis and epicarditis, or bacteriaemia (Jonker et al. 2023; Moeller Jr 2001; Szeredi et al. 2006), resulting from hematogenous or ascending infection routes (Moeller Jr 2001).

##### 3.2.2.6 Leptospira

*Leptospira* spp. have been associated with abortions occurring in the second (Moeller Jr 2001) and third trimesters (Aymée et al. 2022). Pathological findings included serosanguineous abdominal effusion, organ swelling and multifocal haemorrhages in the foetuses (Aymée et al. 2022; Islam et al. 2022; Sharma et al. 2017), although one case showed no gross lesions (Moeller Jr 2001). Histologically, hepatocellular degeneration and interstitial nephritis were common features (Islam et al. 2022; Sharma et al. 2017). Affected does showed no clinical signs (Aymée et al. 2022). Identified species included *Leptospira interrogans* ( *L. interrogans* ) (Islam et al. 2022), *L. noguchii* (Aymée et al. 2022), and *L. pomona* (Moeller Jr 2001).

##### 3.2.2.7 Listeria

The prevalence of *Listeria* spp. ranges from 1% to 6.5% (Chanton‐Greutmann et al. 2002; Engeland et al. 1998; Esmaeili et al. 2025; Moeller Jr 2001; van den Brom et al. 2012). *Listeria* spp. are reported in some studies as a common cause of abortion (van den Brom et al. 2012; van Engelen et al. 2014), though others consider them rare (Hazlett et al. 2013). Abortions occurred in the second and third trimesters (British Veterinary Association 2018; Ismael et al. 2009; Moeller Jr 2001), and were more often linked to *Listeria monocytogenes* ( *L. monocytogenes* ) than to *L. ivanovii* (van den Brom et al. 2012). Lesions vary from none (Szeredi et al. 2006) to necrotizing hepatitis, splenitis, or purulent placentitis (Islam et al. 2022; Ismael et al. 2009; Moeller Jr 2001; van den Brom et al. 2012), while *L. ivanovii* is also associated with necropurulent placentitis and vasculitis, and foetal bronchopneumonia (Moeller Jr 2001; van den Brom et al. 2012). Poor‐quality feed is a potential source of infection (British Veterinary Association 2018; Hussain et al. 1996; Ismael et al. 2009).

##### 3.2.2.8 Mycoplasma

Evidence of *Mycoplasma* has been reported in 1%–1.7% of aborted tissues (Esmaeili et al. 2025; Heidari et al. 2018; Moeller Jr 2001). Pregnancy loss related to *Mycoplasma mycoides* ssp. *mycoides* large colony type was documented in second (Moeller Jr 2001) and third trimesters (Rodríguez et al. 1995). Reported pathological findings in foetuses range from an absence of lesions (Moeller Jr 2001) to pneumonia, and affected does exhibited pneumonia, mastitis, and arthritis (Rodríguez et al. 1995). Detection of *Mycoplasma agalactiae* in aborted foetuses was linked to contagious agalactiae in the dams (Heidari et al. 2018), whereas does aborting *Candidatus Mycoplasma haemobos*‐positive foetuses showed fever, depression and pale mucosae (Shi et al. 2023).

##### 3.2.2.9 Salmonella

*Salmonella* spp. were detected in 1.1% of abortion outbreaks in goat herds in Iran (Esmaeili et al. 2025), although *Salmonella enterica* ( *S. enterica* ) was detected in 1.8% of caprine abortion samples (Ramo et al. 2022). Detailed accounts of *S. enterica* ‐associated abortions are limited, but several serovars have been reported, including *S. enterica* ssp. *arizonae* (Jonker et al. 2023), *S. enterica* ssp. *diarizonae* (Schnydrig et al. 2018), *Salmonella* Abortusovis (Navarro et al. 2009), *Salmonella* Dublin (Atwa and Rady 2007), and *Salmonella Typhimurium* (Atwa and Rady 2007; Jonker et al. 2023; van Engelen et al. 2014). Foetuses infected with *S. enterica* ssp. *arizonae* presented with pulmonary haemorrhages and hepatic necrosis, whereas placentae affected by *Salmonella Typhimurium* showed necrotizing placentitis (Jonker et al. 2023).

##### 3.2.2.10 Staphylococcus

*Staphylococcus* ssp. have been identified in 0.6% of caprine abortion cases (van den Brom et al. 2012). A case reported by Piva et al. 2021 involved a malpositioned foetus aborted due to *Staphylococcus aureus* infection, resulting in the death of the doe. Pathological examination revealed purulent metritis and cotyledonary inflammation. A buck from which a similar isolate was recovered was considered a potential source of infection. In contrast, *Staphylococcus equorum* was associated with abortions presenting no gross lesions (Di Blasio et al. 2019).

##### 3.2.2.11 *Trueperella pyogenes*

*Trueperella pyogenes* , formerly *Arcanobacterium pyogenes* , has been detected in 0.6%–1% of caprine abortions (Moeller Jr 2001; van den Brom et al. 2012), with two reported cases occurring in the second and third trimesters. As no gross lesions were observed, it has been hypothesised that maternal bacteremia led to rapid foetal death (Moeller Jr 2001).

##### 3.2.2.12 Yersinia

*Yersinia* spp. have been detected in 0.5%–2.9% of caprine pregnancy losses (Cho et al. 2025; Esmaeili et al. 2025; van den Brom et al. 2012). Cases were often seasonally clustered in winter and spring, occurring as late‐ or full‐term abortions (Cho et al. 2025; Giannitti et al. 2014). *Yersinia pseudotuberculosis* ( *Y. pseudotuberculosis* ) has been more frequently identified than *Y. enterocolitica* (Cho et al. 2025). Pathological examination of affected foetuses and placentae typically revealed necropurulent pneumonia, hepatitis and placentitis (Cho et al. 2025; Giannitti et al. 2014; van den Brom et al. 2012).

##### 3.2.2.13 Miscellaneous Bacterial Abortifacients

*Aeromonas* sp. was isolated from placenta and foetus in a single case of caprine third‐trimester abortion. No gross lesions were observed, but severe neutrophilic placentitis involving the cotyledonary villi was present, likely resulting from bacteremia or retrograde infection (Moeller Jr 2001).

Evidence of *Anaplasma phagocytophilum* infection was associated with pregnancy loss in goats, with PCR detecting the pathogen in foetal stomach contents, placental tissues, and maternal blood. No gross or histological lesions were observed (Chochlakis et al. 2020).

*Bacillus licheniformis* was detected in five cases of caprine abortion (Hazlett et al. 2013; van den Brom et al. 2021, 2012).

In a single aborted goat foetus, the detection of *Clostridium perfringens* was suggested to be associated with abortion (Waldeland and Løken 1991).

Mainly vectored by ticks, *Ehrlichia canis* has been found in brain tissues of an aborted foetus (Chisu et al. 2021).

*Fusobacterium* sp. was identified in a late‐term abortion with fibrinopurulent placentitis and purulent bronchopneumonia, likely resulting from bacteremia or retrograde infection (Moeller Jr 2001).

*Klebsiella* spp. were identified as the abortifacient agents in three caprine foetuses in Iran (Omidi 2015).

A 3‐week premature aborted twin foetus in an apparently healthy doe showed no gross lesions, but histology revealed pyogranulomatous bronchopneumonia. *Nocardia farcinica* was identified as the predominant pathogen (Vogel et al. 2024).

*Streptococcus* spp. were detected in stomach contents of aborted foetuses, while no gross lesions were observed (Chisu et al. 2021; Szeredi et al. 2006).

#### 3.2.3 Parasitic Pathogens

Parasitic infections were detected in 0.8% (Esmaeili et al. 2025) and 4% of pregnancy losses in goats (Moeller Jr 2001).

##### 3.2.3.1 Neospora caninum

*Neospora caninum* (*N. caninum*) has been identified in 0.6% (Ramo et al. 2022) and 1% (Moeller Jr 2001) of caprine abortion cases. Abortions typically occurred during the second and third trimesters (Campero et al. 2018; Costa et al. 2014; Dubey et al. 1996; Eleni et al. 2004; Mesquita et al. 2018, 2013; Moeller Jr 2001; Regidor‐Cerrillo et al. 2020; Shahiduzzaman et al. 2024). Gross lesions ranged from no visible abnormalities (Barr et al. 1992; Costa et al. 2014; Mesquita et al. 2018) to hydrocephalus, subcutaneous edema, and hepatomegaly (Dubey et al. 1996). Histopathological findings included necrosis, gliosis, and mineralisation in the brain, frequently accompanied by cysts (Barr et al. 1992; Costa et al. 2014; de Oliveira Junior et al. 2020; Dubey et al. 1996; Eleni et al. 2004; Irehan et al. 2022). Parasite cysts have also been detected in the myocardium (Barr et al. 1992; Dubey et al. 1996; Mesquita et al. 2013) and kidneys (Moeller Jr 2001). Additional lesions included myocarditis, hepatitis, pneumonia, and nephritis (Campero et al. 2018; Dubey et al. 1996; Irehan et al. 2022; Moeller Jr 2001). Multifocal necrosis in placental cotyledons was observed in abortion cases of seropositive goats, whereas *N. caninum*‐positive goats that delivered healthy kids exhibited only mild placental lesions, suggesting that impaired oxygen transport may lead to abortion (Mesquita et al. 2018). In one case, an affected doe showed neurological signs characterised by hindlimb paresis (Costa et al. 2014). Infection was transmitted vertically via placenta (Nunes et al. 2017) and persisted across generations (de Oliveira Junior et al. 2020). Dogs were considered a risk factor for infection (Shahiduzzaman et al. 2024).

##### 3.2.3.2 Sarcocystis

*Sarcocystis* schizonts were detected in an aborted caprine foetus from a herd in which eight of 38 does aborted within a 3‐month period. Despite advanced autolysis of the carcass, no gross lesions were observed, whereas histopathological examination revealed multifocal necrotizing non‐purulent encephalitis associated with endothelial‐tropic schizonts. Additional schizonts were identified in the lungs, liver, and glomeruli. Affected does showed vaginal discharge lasting up to 1 week (Mackie et al. 1992). In a subsequent study, the schizonts were confirmed as *Sarcocystis*, most likely *Sarcocystis capracanis* (Mackie and Dubey 1996).

##### 3.2.3.3 *Theileria* spp.

*Theileria lestoquardi* has been shown to cross the placental barrier and induce or contribute to pregnancy loss, as confirmed by microscopy of peripheral blood and PCR of placentomes, the doe's liver, and foetal spleen. The affected doe exhibited inappetence, fever, anaemia, and tick infestation (Esmaeilnejad et al. 2018). In contrast, *Theileria sergenti/orientalis/buffeli* was detected only in coinfections (Chisu et al. 2021).

##### 3.2.3.4 Toxoplasma gondii

Reported as the second (Chanton‐Greutmann et al. 2002; van Engelen et al. 2014) or third most important infectious cause of abortion in goats (Alzuguren et al. 2023; Ramo et al. 2022), *Toxoplasma gondii* (*T. gondii*) has been detected in 0.71% (Waldeland and Løken 1991) to 15% (Chanton‐Greutmann et al. 2002) of cases. Reported abortion rates in affected herds ranged from 11% (Giadinis et al. 2013) to 54% (Gufler et al. 1999), with outbreaks occasionally affecting entire groups of pregnant does (Vilela et al. 2024). Abortions typically occurred during the second and third trimester (Amouei et al. 2019; de Oliveira et al. 2018; Giadinis et al. 2013; Gufler et al. 1999; Hasan et al. 2021; Moeller Jr 2001; Partoandazanpoor et al. 2020; Pereira et al. 2021; Silva Filho et al. 2008; Vilela et al. 2024; Waldeland and Løken 1991), and live‐born kids often died shortly after birth (Bari et al. 1993; Skinner et al. 1990). Clinical signs in affected does included reduced milk production, inappetence, dehydration, vaginal discharge, and fever (de Oliveira et al. 2018; Gufler et al. 1999; Skinner et al. 1990). Following abortion, foetid vaginal discharge, retained placenta, and elevated body temperature were observed (de Oliveira et al. 2018; Gufler et al. 1999). Gross lesions varied from no visible abnormalities (Gufler et al. 1999; Moreno et al. 2012; Partoandazanpoor et al. 2020) to foetal anasarca, autolysis, and necrotizing placentitis (Bari et al. 1993; Gufler et al. 1999; van den Brom et al. 2012). Microscopically, necrotizing encephalitis, hepatitis, myocarditis, and myositis have been reported (de Oliveira et al. 2018; Irehan et al. 2022; Islam et al. 2022; Pereira et al. 2021), along with necrotizing placentitis affecting the cotyledons and placental mineralisation (Al‐Mufarrej et al. 2011; Bari et al. 1993; Caldeira et al. 2011; Chanton‐Greutmann et al. 2002; Moeller Jr 2001; Partoandazanpoor et al. 2020; van den Brom et al. 2012). Lesions in brain and placental tissue were most frequently observed (Bari et al. 1993; Hasan et al. 2021), although confirmation in other tissues was also recommended (Masala et al. 2003; Vilela et al. 2024). The presence of cats has been considered a major risk factor for *T. gondii*‐associated abortions (Hasan et al. 2021; Pereira et al. 2021; Vilela et al. 2024), whereas Alpine farming was identified as a protective factor (Basso et al. 2022). Sexual transmission has also been described (Santana et al. 2013).

##### 3.2.3.5 Trypanosoma vivax

*Trypanosoma vivax* ( *T. vivax* ) has been identified as the causative agent of abortion in a Brazilian herd experiencing trypanosomiasis, with a high incidence of late‐term pregnancy losses (53.8%). Abortions occurred predominantly in does with high levels of parasitemia. Affected animals exhibited depression, inappetence, fever, and pallor. Pathological examination revealed necrotizing hemorrhagic placentitis involving the cotyledons, a swollen umbilical cord, foetal anasarca, ascites, and haemorrhages in various foetal organs. Histologically, multifocal lymphoplasmacytic hepatitis, nephritis and encephalitis were observed. PCR confirmed *T. vivax* infection in foetal tissues, indicating vertical transmission secondary to maternal parasitemia (Batista et al. 2022).

#### 3.2.4 Fungal Pathogens and Toxins

Fungal agents were detected in 0.5% (Moeller Jr 2001) to 1% of caprine abortion cases (Esmaeili et al. 2025).

*Aspergillus* mycotoxicosis was identified as a cause of late‐term abortion in four of nine pregnant goats presenting with anaemia and neutropenia. The fungus was isolated from both feed and foetal stomach contents, suggesting ingestion of contaminated feed as the source of transplacental infection. Secondary infections were also observed (Biobaku et al. 2016). Aflatoxin‐induced abortions and neonatal deaths were reported in a goat herd of 300 animals, where 15 abortions at various gestational stages and three neonatal deaths occurred. Affected does were showing drowsiness, inappetence, diarrhoea, and a dry muzzle. Gross examination of aborted foetuses revealed icterus and pale, friable livers with haemorrhages. Histologically, fatty liver, bile ductular hyperplasia, catarrhal gastroenteritis, and focal nephrosis were observed. Aflatoxin B_1_ residues were detected in foetal livers (10–20 ppb), gingelly oilcake (2000 ppb), and maize (400 ppb) (Maryamma et al. 1990). *Aspergillus fumigatus* (Atwa and Rady 2007; Esmaeili et al. 2025; Omidi 2015) and *Aspergillus niger* (Atwa and Rady 2007) have been reported in additional cases.

A case of late‐term abortion associated with *Candida albicans* showed mycotic bronchopneumonia and intercotyledonary placentitis (Moeller Jr 2001).

Other fungal species implicated in pregnancy loss include *Candida tropicalis* , *Mucor* spp., and *Rhizopus* spp. (Atwa and Rady 2007).

#### 3.2.5 Coinfections

Coinfections were reported in 22 publications, with *Coxiella burnetii* ( *C. burnetii* ) being the pathogen most frequently mentioned in combination with other infectious agents (*n* = 12) (Alzuguren et al. 2023; Chisu et al. 2021; Esmaeili et al. 2025; Hazlett et al. 2013; Jonker et al. 2023; Kreizinger et al. 2015; Ramo et al. 2022; Santos et al. 2022; Schöpf et al. 1991; Şevik 2024; Twomey et al. 2011; van den Brom et al. 2012). The most frequent combination involved *C. burnetii* and *Chlamydia* spp. (*n* = 11) (Alzuguren et al. 2023; Chisu et al. 2021; Esmaeili et al. 2025; Hazlett et al. 2013; Jonker et al. 2023; Kreizinger et al. 2015; Ramo et al. 2022; Santos et al. 2022; Schöpf et al. 1991; Şevik 2024; Twomey et al. 2011), particularly *C. burnetii* and *Chlamydia abortus* (*n* = 6) (Alzuguren et al. 2023; Chisu et al. 2021; Hazlett et al. 2013; Ramo et al. 2022; Şevik 2024; Twomey et al. 2011). In one study, 44 of 148 *C. burnetii* –positive abortion cases were mixed infections with *Chlamydia abortus* (Ramo et al. 2022).

*Toxoplasma gondii* was also identified in coinfections, most commonly with *Neospora caninum* (*n* = 3) (Irehan et al. 2022; Masala et al. 2007; Unzaga et al. 2014), or *C. burnetii* (*n* = 3) (Alzuguren et al. 2023; Hazlett et al. 2013; Ramo et al. 2022). While most reported coinfections involved at least one established abortifacient, abortion during a shipping fever outbreak appeared to result primarily from septicemia, in which *Mannheimia haemolytica* and *Pasteurella multocida* were detected. As shipping fever is a multifactorial disease, additional abortigenic factors should be considered (Karabasanavar et al. 2016).

### 3.3 Non‐Infectious Agents

Non‐infectious agents were identified in 1% (1 of 75) (Szeredi et al. 2006), 10% (Moeller Jr 2001) and 15.2% (Esmaeili et al. 2025) of caprine abortion cases.

#### 3.3.1 Maternal, Management‐Related and Iatrogenic Causes

##### 3.3.1.1 Metabolic and Nutritional Causes

###### 3.3.1.1.1 Pregnancy Toxaemia

Pregnancy toxaemia (PT) accounted for 4.8% of caprine pregnancy losses (Esmaeili et al. 2025). On a dairy goat farm comprising 1800 animals, 32 does were diagnosed with PT based on inappetence and blood β‐hydroxybutyric acid (BHBA) concentrations ≥ 3 mmol/L. Clinical signs included polypnea, limb edema, neurological disturbances and ketonuria. Depending on blood pH, pregnancy was terminated by intramuscular administration of dexamethasone (1 mg/10 kg) and dexcloprostenol (125 μL) or by caesarean section. All does undergoing caesarean section died, whereas eight of 22 induced does survived. Survival was associated with inappetence but preserved ruminal motility, while recumbency, aciduria and altered blood chemistry (pH, pCO₂, HCO₃^−^, BE, K^+^) were linked to death. BHBA concentration had no prognostic relevance. Early pregnancy termination was recommended to increase the likelihood of viable offspring (Lima et al. 2016).

Pregnancy toxaemia has also been experimentally induced through restricted energy intake, causing weakness, apathy, incoordination, recumbency, teeth grinding and abortion (Affan et al. 2022; Zainal Ulum et al. 2021). Pathological findings included umbilical cord desquamation, placental microthrombosis, haemorrhage, congestion, and chorionic villous immaturity, as well as foetal hepatic necrosis. Elevated BHBA Vogt and hypoglycemia were associated with abortion (Affan et al. 2022; Zainal Ulum et al. 2021), along with decreased insulin, calcium, and potassium, and increased cortisol and free fatty acids (Affan et al. 2022).

###### 3.3.1.1.2 Vitamin E/Selenium Deficiency

Vitamin E and selenium deficiencies have been associated with 2% (Chanton‐Greutmann et al. 2002) to 7.5% of caprine abortion cases (Esmaeili et al. 2025). Selenium deficiency‐related abortions occurred primarily in the third trimester, and one of six affected foetuses exhibited myocardial degeneration and necrosis. Low hepatic selenium concentrations (0.019–0.187 ppm) were commonly observed (Moeller Jr 2001).

###### 3.3.1.1.3 Copper Deficiency

Copper deficiency was identified as the cause of abortion in two cases, representing 1% of caprine abortions. The abortions occurred during the last trimester, and histological examination revealed demyelination of the white matter. Low copper concentrations (1.6–6.0 ppm) were diagnosed (Moeller Jr 2001).

##### 3.3.1.2 Dietary Manipulations

###### 3.3.1.2.1 Nutritional Imbalances and Feed Quality

In a feeding trial comparing hay and silage of varying quality and energy levels, plasma progesterone concentrations were lowest in goats receiving poor‐quality silage and 70% of maintenance energy, resulting in abortions following a rapid decline of progesterone to near zero (Hussain et al. 1996). Reproductive losses were highest in does fed poor‐quality, low‐energy silage, particularly among older animals, whereas adequate roughage quality supported good reproductive performance irrespective of energy level. Abortions occurred predominantly in the third trimester (Hussain et al. 1996).

Reduced energy intake during mid‐gestation led to maternal weight loss and 15% higher foetal losses, emphasising the need to minimise weight loss during this period (McGregor 2016). In another study, aborted does had lower crude protein intake and consumed more fibrous diets compared with non‐aborted does, suggesting undernutrition and reduced dietary selectivity as contributing factors (Mellado 2014). Supplementary feeding with multipurpose leguminous trees such as *Calliandra calothyrsus* and *Leucaena leucocephala* reduced abortion rates and improved maternal body weight (Pamo et al. 2006), despite the known toxicity of *Leucaena leucocephala* (Sastry and Singh 2008).

###### 3.3.1.2.2 Experimental Toxin Exposure

Experimental administration of *Claviceps purpurea* sclerotia containing ergotamine via stomach tube to pregnant goats resulted in progressive deterioration, fever, inappetence, body weight loss, depression, and abortions occurring 33 and 47 days after exposure, corresponding to gestational days 136 and 140. Elevated prostaglandin F_2_α concentrations in aborted goats indicated a disruption of endocrine foetal–placental function (Vogt Engeland et al. 1998).

Toxic plant‐feeding experiments in pregnant goats, conducted mainly in Brazil, have demonstrated their abortifacient potential.

*Amorimia septentrionalis*, a monoflouroacetate‐containing plant, caused depression, tachycardia, tachypnea, tremors, ataxia and recumbency, leading to abortions and the birth of live kids that often died shortly after birth or following colostrum intake. Major lesions included pulmonary edema, hepatocellular degeneration and hydropericardium (Da Silva et al. 2017; Lopes et al. 2019).

*Leucaena leucocephala* , rich in mimosine, induced abortions proportional to daily intake, with foetuses showing parenchymatous and colloidal goitre (Sastry and Singh 2008), although lower abortion rates have been reported when used as a leguminous supplement (Pamo et al. 2006).

Ingestion of *Mimosa tenuiflora* (Dantas et al. 2012) and *Poincianella pyramidalis* resulted in embryonic loss and abortion (Santos et al. 2018; Santos Dos Reis et al. 2016).

Does ingesting *Stryphnodendron fissuratum* exhibit apathy, anorexia, ruminal hypomotility, jaundice, ataxia, lateral recumbency, abortions and depending on intake level (Albuquerque et al. 2011).

Abortions following *Tetrapterys multiglandulosa* exposure were linked to foetal malnutrition, with underdeveloped, autolytic foetuses showing haemorrhages in skin, meninges and serosae (Melo et al. 2001).

##### 3.3.1.3 Medical Interventions

###### 3.3.1.3.1 Prostaglandin F_2_α

Prostaglandin F_2_α (PGF_2_α) has been administered intramuscularly to pregnant goats to induce abortion. In one Saanen goat and three Himalayan tahrs weighing 35–48 kg, 15 mg PGF_2_α was injected, resulting in abortion within 40–52 h and a marked decline in progesterone concentrations immediately after administration (Yong et al. 2010). Six Sokoto Red goats received 7.5 mg PGF_2_α, which led to abortion in all animals within 72.0 ± 12.4 h, accompanied by blood‐tinged vaginal discharge (Kawu et al. 2013). In a study of fifteen pregnant Boer goats at 60 days of gestation, animals were given 0.2 mg PGF_2_α, followed by 0.1 mg at 24‐h intervals, resulting in abortion in all does within 36–48 h. Affected does exhibited swelling and reddening of the labium, and bloody vaginal mucus (Chen et al. 2016).

###### 3.3.1.3.2 Administration of Selenium

Acute neonatal selenosis was induced by parenteral selenium and vitamin E administration, despite maternal supplementation. Newborns showed severe dyspnea and incoordination and died within 8 h. Gross findings included pulmonary edema, hydrothorax and hydropericardium, while histology confirmed acute myocardial contraction band necrosis (Amini et al. 2011).

###### 3.3.1.3.3 Vaccination

Vaccination with live attenuated pathogens has been reported to induce abortion in goats. Conjunctival administration of *Brucella melitensis* ( *B. melitensis* ) Rev. 1 led to late‐term abortion (Zundel et al. 1992), as did subcutaneous vaccination with *B. melitensis* RB51, rfbK and Rev. 1 strains (Villa et al. 2008). Following vaccination with a Rift Valley fever virus (RVFV) vaccine, goats developed sudden, severe fever up to 41°C and pregnancy loss occurring 10–28 days later (Kamal 2009). Late‐term abortions were also reported 24–29 days after a double dose of goatpox vaccine, with affected does exhibiting fever and pox lesions (Esmaeili et al. 2024).

#### 3.3.2 Foetal Causes

##### 3.3.2.1 Developmental Abnormalities

###### 3.3.2.1.1 Congenital Malformations

Foetal malformations accounted for 0.6% (Esmaeili et al. 2025; van den Brom et al. 2012) and 3% (Chanton‐Greutmann et al. 2002; Moeller Jr 2001) of pregnancy losses in goats. Reported anomalies included leukoencephalomalacia, scoliosis, arthrogryposis, cyclopia, and hydrocephalus, typically resulting in abortion during the third trimester (Moeller Jr 2001).

###### 3.3.2.1.2 Foetal Thyroid Hyperplasia

Foetal thyroid hyperplasia accounted for 1.5% (Moeller Jr 2001) and 1.6% (Esmaeili et al. 2025) of caprine pregnancy losses and was associated with multifocal myocardial necrosis in third‐trimester aborted foetuses. Iodine concentrations were not assessed (Moeller Jr 2001), and a thyroid‐to‐body weight ratio equal to or exceeding 0.40 g/kg was considered diagnostic for goitre (Esmaeili et al. 2025).

##### 3.3.2.2 Mechanical and Traumatic Causes

###### 3.3.2.2.1 Umbilical Cord Torsion

At 135 days of gestation, a doe exhibiting lethargy, congested mucous membranes, vulvar edema, and continuous vaginal discharge aborted a foetus by assistance. Subsequently, a mummified foetus aged 6–8 weeks was delivered, showing severe umbilical cord torsion of 720° with an evident abdominal indentation. Histopathological examination revealed a constricted lumen of the umbilical vessels, indicating foetal death due to ischemia (Sobanaasree et al. 2017).

###### 3.3.2.2.2 Uterine Torsion

A dead kid was expelled following a post‐cervical, left‐sided uterine torsion. Detorsion was performed by tightly tying a rope around the doe's abdomen and rolling her rapidly and steadily towards the side of the torsion (Jakkali et al. 2022).

###### 3.3.2.2.3 Foetal Trauma

Trauma was identified as the cause of pregnancy loss in three of 623 abortion cases. The affected full‐term foetuses exhibited cranial haemorrhage, rib fractures, intrathoracic haemorrhage and hepatic rupture (Esmaeili et al. 2025).

### 3.4 Unknown Agents

In 41.7% (Chanton‐Greutmann et al. 2002) to 60.3% (Esmaeili et al. 2025) and 65.9% (van den Brom et al. 2012) of examined aborted goat foetuses, no definitive diagnosis could be established, whereas a cause was identified in 65.6% of abortion outbreaks in goat herds (Esmaeili et al. 2025).

In 10% (van den Brom et al. 2012) to 21.5% of cases, lesions such as placentitis suggested an infectious aetiology, although no causative agent could be detected (Chanton‐Greutmann et al. 2002).

## 4 Discussion

This scoping review provides an overview of the existing literature on infectious and non‐infectious causes of pregnancy loss in goats published in English, German and French since 1990.

Across the 203 included studies, a wide range of abortifacients was identified, although the causative agent remains unknown in a substantial proportion of cases. Many studies reported clinical signs in the doe, pathological changes, and management or housing conditions, but this information was inconsistently documented, limiting systematic analysis of abortifacient‐specific patterns. Experimental infection studies often provide more detailed information; however, they were excluded to focus on naturally occurring cases and practical relevance.

Most pregnancy losses occurred during the second and third trimesters, reflecting both the greater clinical visibility of late‐term abortion compared with early foetal loss and the exclusion of studies focusing solely on embryonic mortality. Pregnancy loss was most frequently associated with infectious agents, particularly bacteria. *B. melitensis* , *Chlamydia abortus*, *C. burnetii* , *Listeria monocytogenes* , and the protozoan *Toxoplasma gondii* were among the most commonly detected pathogens, broadly paralleling with the number of studies addressing them. However, this pattern may also reflect the availability of diagnostic assays, their zoonotic relevance, and established surveillance structures. Some pathogens may be underrepresented due to omission from routine molecular panels or limited research attention.

Studies on non‐infectious causes, such as nutritional imbalances, plant intoxications, metabolic disorders, congenital abnormalities, and mechanical factors were less frequently reported, reflecting diagnostic complexity, multifactorial aetiology, individual influences and the prioritisation of infectious causes in routine investigations. The assessment of suspected mineral deficiencies is further limited by the scarcity of validated reference ranges for abortion samples, restricting interpretative reliability. Additionally, potentially abortigenic toxic substances may remain undetectable at the time of sampling. Even when comprehensive post‐mortem examinations are performed, non‐infectious etiologies often lack specific or pathognomic lesions. Moreover, these conditions are frequently documented in sporadic cases or small case series, limiting diagnostic investigation (Esmaeili et al. 2025).

Most included studies were published within the past 15 years. This upward trend may reflect increasing scientific attention to caprine reproductive health, improved diagnostic capacity, or a growing awareness of the economic and public health implications of pregnancy loss in goats. Conversely, the lower number of earlier publications may partly result from limited digital accessibility or restricted diagnostic resources.

The geographical distribution of included studies did not fully correspond to global goat population density. While approximately 50% of the global goat population is located in Asia and about 45% in Africa (FAOSTAT Database 2025), 37% of the included studies originated from Asia (*n* = 75), 28% from Europe (*n* = 57), and 27% from the Americas (*n* = 54). The comparatively low representation of African countries likely reflects disparities in access to veterinary infrastructure, diagnostic services, financial resources and surveillance systems rather than true differences in disease occurrence (Assefa et al. 2024; Semango and Buza 2024). Limited surveillance capacity in resource‐constrained regions may further contribute to underreporting of pregnancy loss and its etiologies (Dubey et al. 2020; Semango and Buza 2024).

Goats represent an economically and nutritionally important livestock species worldwide, particularly for smallholder farmers in low‐ and middle‐income countries (Airs et al. 2023; Dossa et al. 2008; Dubey et al. 2020; Kaumbata et al. 2020; Miller et al. 2012; Sone et al. 2015). Abortion is associated not only with the loss of offspring but also with decreased milk yield, impaired fertility, increased culling rates, additional veterinary costs, and substantial economic burdens at both herd and national levels (Gutiérrez‐Expósito et al. 2021; Lokamar et al. 2020; Margatho et al. 2019; Ngetich 2024; Rossetti et al. 2017; Semango et al. 2024; Stelzer et al. 2019). Consequently, pregnancy loss constitutes a significant challenge for animal health, farm profitability, and food security, particularly in economically vulnerable production systems. Strengthening preventive health measures, reproductive management, and applied field research may substantially contribute to reducing its impact (Airs et al. 2023; Esmaeili et al. 2025; Martins and Lilenbaum 2014).

Publication delays following abortion events were evident in several outbreaks, notably those involving Schmallenberg virus, bluetongue virus and other arboviruses, where peer‐reviewed reports appeared after a substantial delay. Such temporal gaps limit the utility of the published literature for real‐time decision‐making during emerging disease events.

As recommended for scoping reviews, no rating of the quality of evidence was provided.

Percentages were reported only for sample sizes large enough to support meaningful interpretation; otherwise, absolute numbers were used.

## 5 Conclusion

The findings of this scoping review indicate that pregnancy loss in goats remains a multifactorial and diagnostically challenging condition, with the underlying cause remaining unconfirmed in a substantial proportion of cases. This highlights the need for standardised diagnostic procedures, more comprehensive and systematic investigation of pregnancy loss in goats, increased attention to non‐infectious causes, and improved documentation of relevant herd‐ and management‐related factors. Such efforts are essential to reduce the high proportion of pregnancy losses with unidentified aetiology. At the same time, the role of goats has changed markedly. Rather than being regarded primarily as environmental destroyers, goats are increasingly recognised as valuable contributors to milk production in developed countries and as important assets for poverty alleviation and livelihood security in less developed countries. In this context, applied research, particularly in the field of pregnancy loss, has strong potential to support the sustainable development and genetic improvement of this important livestock species. Promoting interdisciplinary collaboration and applied herd‐level research would enhance our understanding of pregnancy loss in goats and strengthen efforts to improve reproductive efficiency, animal welfare, public health and the mitigation of associated socio‐economic consequences.

## Author Contributions

**Friederike Maria Kaus:** writing – original draft, methodology, investigation, data curation, conceptualization, visualisation. **Hannah Eggimann:** methodology. **Barbara Tschulena:** methodology. **Gaby Hirsbrunner:** writing – review and editing, supervision, methodology, conceptualization. **Patrik Zanolari:** writing – review and editing, supervision, methodology, conceptualization.

## Funding

The authors have nothing to report.

## Conflicts of Interest

The authors declare no conflicts of interest.

## Supporting information


**Appendix S1:** rda70198‐sup‐0001‐AppendixS1.pdf.

## Data Availability

The data that supports the findings of this study are available in the Supporting Information of this article.
