# Supplementary material for: A Review of Infectious and Non‐Infectious Causes of Pregnancy Loss in Goats
Source: Reprod Domest Anim. 2026 Mar 24;61(3):e70198. doi: 10.1111/rda.70198 (PMC13014034; doi:10.1111/rda.70198)
Supplement: Supplementary file 1 — Appendix S1: rda70198‐sup‐0001‐AppendixS1.pdf. [file RDA-61-e70198-s001.pdf]

Table 1 Infectious agents causing abortions

**Group**		**Infectious agent**		**Citation**
Viruses	Flaviviridae	BDV		(Sharawi et al., 2010)
				(Toplu et al., 2011)
				(Rosamilia et al., 2014)
				(Ramo, María de Los Angeles et al., 2022)
				(Esmaeili et al., 2025)
		BVDV		(Broaddus et al., 2009)
				(Lamm et al., 2009)
			BVDV 3 (HoBi-like pestivirus)	(Shi et al., 2023)
		Pestiviruses		(Albayrak & Özan, 2012)
				(Tuncer-Göktuna et al., 2016)
				(Golender et al., 2021)
				(Şevik, 2021)
				(Akman & Okur Gumusova, 2023)
				(Alzuguren et al., 2023)
	Herpesviridae	CpHV		(Williams et al., 1997)
				(Moeller, 2001)
			CpHV 1	(Chénier et al., 2004)
				(Uzal et al., 2004)
				(McCoy et al., 2007)
				(Gonzalez et al., 2017)
	Paramyxoviridae	PPRV		(Pestil et al., 2020)
				(Murat, 2024)
	Peribunyaviridae	AKAV		(Golender et al., 2021)
				(Cagirgan et al., 2022)
		CVV		(Edwards et al., 2003)
		Schmallenberg		(Herder et al., 2012)
				(Dominguez et al., 2014)
				(Wagner et al., 2014)
		SHAV		(van der Walt et al., 2023)
		SHUV		(Golender et al., 2021)
				(Golender et al., 2022)
	Sedoreoviridae	BTV		(Chauhan et al., 2014)
				(Golender et al., 2020)
				(Golender et al., 2021)
		BTV breakthrough infection		(Savini et al., 2014)
Bacteria		*Brucella*		(Maksimović et al., 2022)
			Brucella abortus, B. melitensis	(Ntivuguruzwa et al., 2022)
			Brucella abortus, B. ovis	(Alirezaei et al., 2024)
			Brucella melitensis	(Ribeiro et al., 1990)
				(Aldomy et al., 1992)
				(Al-Ani et al., 2004)
				(Al-Majali, 2005)
				(Atwa & Rady, 2007)

			(Büyükcangaz et al., 2009)
			(Samadi et al., 2010)
			(Sanjay Ghodasara et al., 2010)
			(Moshkelani et al., 2011)
			(Palanivel et al., 2012)
			(Singh et al., 2013)
			(Abnaroodheleh et al., 2021)
			(Dadar & Alamian, 2021a)
			(Dadar & Alamian, 2021b)
			(Behroozikhah et al., 2022)
			(Dadar et al., 2022)
			(Demirpence et al., 2022)
			(Katsiolis et al., 2022)
			(Dadar et al., 2023)
			(Dadar & Alamian, 2025)
			(Esmaeili et al., 2025)
	Campylobacter		(van den Brom et al., 2012)
			(Díaz-Cao et al., 2018)
			(van Brom et al., 2021)
			(Ramo, María de Los Angeles et al., 2022)
			(Alzuguren et al., 2023)
			(Esmaeili et al., 2025)
		Campylobacter fetus	(Omidi, 2015)
			(Sakmanoğlu et al., 2021)
		Campylobacter fetus ssp. *fetus*	(Atwa & Rady, 2007)
		Campylobacter fetus ssp. *venerealis*	(Atwa & Rady, 2007)
		Campylobacter jejuni	(Sahin et al., 2012)
		Campylobacter jejuni ssp. *jejuni*	(Moeller, 2001)
			(Scarcelli et al., 2009)
			(Jonker et al., 2023)
	Chlamydia		(van Engelen et al., 2014)
			(Szeredi et al., 2020)
			(S. Santos et al., 2022)
		Chlamydia abortus	(Wang et al., 2001)
			(Chanton-Greutmann et al., 2002)
			(Szeredi & Bacsadi, 2002)
			(Masala et al., 2005)
			(Szeredi et al., 2006)
			(Navarro et al., 2009)
			(Twomey et al., 2011)
			(van den Brom et al., 2012)
			(Hazlett et al., 2013)
			(Kalender et al., 2013)
			(Ababneh et al., 2014)
			(Campos-Hernández et al., 2014)
			(Kreizinger et al., 2015)

			(Schnydrig et al., 2017)
			(Hailat et al., 2018)
			(Heidari et al., 2018)
			(Di Paolo et al., 2019)
			(Špičić et al., 2019)
			(Chisu et al., 2021)
			(Fayez et al., 2021)
			(Malal & Turkyilmaz, 2021)
			(Islam et al., 2022)
			(Ramo, María de Los Angeles et al., 2022)
			(Alzuguren et al., 2023)
			(Şevik, 2024)
			(Esmaeili et al., 2025)
		Chlamydia abortus, C. pecorum	(Jonker et al., 2023)
		Chlamydia pecorum	(Giannitti et al., 2016)
		Chlamydia psittaci	(Escalante-Ochoa et al., 1997)
			(Liao et al., 1997)
			(Moeller, 2001)
			(Zaitsev et al., 2024)
		Chlamydia psittaci, C. abortus	(Brijesh Bhardwaj et al., 2017)
		Chlamydia psittaci, C. abortus, other	(van Brom et al., 2021)
	Coxiella burnetii		(Copeland et al., 1991)
			(Moore et al., 1991)
			(Sanford et al., 1993)
			(Sanford et al., 1994)
			(Moeller, 2001)
			(Chanton-Greutmann et al., 2002)
			(Masala et al., 2004)
			(Szeredi et al., 2006)
			(Navarro et al., 2009)
			(Jones et al., 2010)
			(Cantas et al., 2011)
			(Pritchard et al., 2011)
			(Twomey et al., 2011)
			(Astobiza et al., 2012)
			(Reichel et al., 2012)
			(van den Brom et al., 2012)
			(Hazlett et al., 2013)
			(Günaydin et al., 2015)
			(Abdel-Moein & Hamza, 2017)
			(Magouras et al., 2017)
			(Schnydrig et al., 2017)
			(Álvarez-Alonso et al., 2018)
			(de Oliveira, Júnior Mário Baltazar, Rozental, et al., 2018)
			(Heidari et al., 2018)

			(Rajagunalan et al., 2019)
			(Heinzelmann et al., 2020)
			(Chisu et al., 2021)
			(Mohabati Mobarez et al., 2021)
			(Saleh et al., 2021)
			(Ozgen et al., 2022)
			(Ramo, María de Los Angeles et al., 2022)
			(S. Santos et al., 2022)
			(Alzuguren et al., 2023)
			(Hemsley et al., 2023)
			(Mangena et al., 2023)
			(Mohabati Mobarez et al., 2023)
			(Borhani et al., 2024)
			(Liu et al., 2024)
			(Şevik, 2024)
			(Esmaeili et al., 2025)
	Escherichia coli		(Moeller, 2001)
			(Szeredi et al., 2006)
			(Omidi, 2015)
			(Jonker et al., 2023)
			(Esmaeili et al., 2025)
	Leptospira		(K. K. Sharma et al., 2017)
		Leptospira interrogans	(Islam et al., 2022)
		Leptospira noguchii	(Aymée et al., 2022)
		Leptospira pomona	(Moeller, 2001)
	Listeria		(Díaz-Cao et al., 2018)
			(van Brom et al., 2021)
			(Esmaeili et al., 2025)
		Listeria monocytogenes	(Waldeland & Løken, 1991)
			(Hussain, Waldeland, et al., 1996)
			(Engeland et al., 1998)
			(Chanton-Greutmann et al., 2002)
			(Szeredi et al., 2006)
			(Atwa & Rady, 2007)
			(Ismael et al., 2009)
			(Hazlett et al., 2013)
			(van Engelen et al., 2014)
			(British Veterinary Association, 2018)
			(Islam et al., 2022)
			(Şevik, 2024)
		Listeria monocytogenes, L. ivanovii	(Moeller, 2001)
			(van den Brom et al., 2012)
	Mycoplasma		(Esmaeili et al., 2025)
		Mycoplasma (Candidatus Mycoplasma haemobos)	(Shi et al., 2023)

		Mycoplasma agalactiae	(Heidari et al., 2018)
		Mycoplasma mycoides ssp. *mycoides* LC	(Rodríguez et al., 1995)
			(Moeller, 2001)
	Salmonella		(Esmaeili et al., 2025)
		Salmonella enterica	(Ramo, María de Los Angeles et al., 2022)
			(Alzuguren et al., 2023)
		Salmonella enterica ssp. *arizonae*	(Jonker et al., 2023)
		Salmonella enterica ssp. *diarizonae*	(Schnydrig et al., 2018)
		Salmonella enterica ssp. *enterica serovar* Abortusovis	(Navarro et al., 2009)
		Salmonella enterica ssp. *enterica serovar* Dublin	(Atwa & Rady, 2007)
		Salmonella enterica ssp. *enterica serovar* Typhimurium	(Atwa & Rady, 2007)
			(van Engelen et al., 2014)
			(Jonker et al., 2023)
	Staphylococcus		(van den Brom et al., 2012)
			(Omidi, 2015)
		Staphylococcus aureus	(Atwa & Rady, 2007)
			(Hazlett et al., 2013)
			(Piva et al., 2021)
		Staphylococcus equorum	(Di Blasio et al., 2019)
	Streptococcus		(Szeredi et al., 2006)
	Trueperella pyogenes		(Moeller, 2001)
			(van den Brom et al., 2012)
			(Hazlett et al., 2013)
			(Esmaeili et al., 2025)
	Yersinia	*Yersinia pseudotuberculosis*	(van den Brom et al., 2012)
			(Giannitti et al., 2014)
			(van Engelen et al., 2014)
		Yersinia pseudotuberculosis, Y. enterocolitica	(Cho et al., 2025)
	Miscellaneous	*Aeromonas*	(Moeller, 2001)
		Anaplasma phagocytophilum	(Chochlakis et al., 2020)
		Bacillus licheniformis	(van den Brom et al., 2012)
			(Hazlett et al., 2013)
			(van Brom et al., 2021)
		Clostridium perfringens	(Waldeland & Løken, 1991)
		Ehrlichia canis	(Chisu et al., 2021)
		Fusobacterium	(Moeller, 2001)
		Klebsiella	(Omidi, 2015)
		Nocardia farcinica	(Vogel et al., 2024)
Parasites	*Neospora*	*Neospora caninum*	(Dubey et al., 1996)
			(Moeller, 2001)

			(Eleni et al., 2004)
			(Masala et al., 2007)
			(Moreno et al., 2012)
			(Mesquita et al., 2013)
			(Costa et al., 2014)
			(Unzaga et al., 2014)
			(Nunes et al., 2017)
			(Schnydrig et al., 2017)
			(Campero et al., 2018)
			(Díaz-Cao et al., 2018)
			(Mesquita et al., 2018)
			(de Oliveira Junior, Ivam Moreira et al., 2020)
			(Regidor-Cerrillo et al., 2020)
			(Basso et al., 2022)
			(Irehan et al., 2022)
			(Ramo, María de Los Angeles et al., 2022)
			(Mohammed et al., 2023)
			(Shahiduzzaman et al., 2024)
		Neospora-like protozoe	(Barr et al., 1992)
	Sarcocystis		(Mackie et al., 1992)
			(Mackie & Dubey, 1996)
	Theileria lestoquardi		(Esmaeilnejad et al., 2018)
	Toxoplasma gondii		(Skinner et al., 1990)
			(Waldeland & Løken, 1991)
			(Bari et al., 1993)
			(Gufler et al., 1999)
			(Moeller, 2001)
			(Chanton-Greutmann et al., 2002)
			(Masala et al., 2003)
			(S. P. Sharma et al., 2003)
			(Masala et al., 2007)
			(Silva Filho, M. de F. et al., 2008)
			(Al-Mufarrej et al., 2011)
			(Caldeira et al., 2011)
			(Moreno et al., 2012)
			(van den Brom et al., 2012)
			(Giadinis et al., 2013)
			(Hazlett et al., 2013)
			(Santana et al., 2013)
			(Unzaga et al., 2014)
			(van Engelen et al., 2014)
			(de Oliveira, Júnior Mário Baltazar, Almeida, et al., 2018)
			(Amouei et al., 2019)
			(Sah et al., 2019)
			(Partoandazanpoor et al., 2020)
			(Hasan et al., 2021)

			(Pereira et al., 2021)
			(Basso et al., 2022)
			(Irehan et al., 2022)
			(Islam et al., 2022)
			(Oliveira et al., 2022)
			(Ramo, María de Los Angeles et al., 2022)
			(Alzuguren et al., 2023)
			(Vilela et al., 2024)
			(Esmaeili et al., 2025)
	Trypanosoma vivax		(Batista et al., 2022)
Fungi	*Aspergillus*		(Biobaku et al., 2016)
		Aspergillus fumigatus	(Atwa & Rady, 2007)
			(Omidi, 2015)
			(Esmaeili et al., 2025)
		Aspergillus niger	(Atwa & Rady, 2007)
	Candida	*Candida albicans*	(Moeller, 2001)
			(Atwa & Rady, 2007)
			(Esmaeili et al., 2025)
		Candida tropicalis	(Atwa & Rady, 2007)
	Mucor		(Atwa & Rady, 2007)
	Rhizopus		(Atwa & Rady, 2007)
Coinfections			(Schöpf et al., 1991)
			(Chanton-Greutmann et al., 2002)
			(Masala et al., 2007)
			(Twomey et al., 2011)
			(van den Brom et al., 2012)
			(Hazlett et al., 2013)
			(Unzaga et al., 2014)
			(Kreizinger et al., 2015)
			(Karabasanavar et al., 2016)
			(Di Blasio et al., 2019)
			(Coe et al., 2020)
			(Abnaroodheleh et al., 2021)
			(Chisu et al., 2021)
			(Sakmanoğlu et al., 2021)
			(Irehan et al., 2022)
			(Ramo, María de Los Angeles et al., 2022)
			(S. Santos et al., 2022)
			(Alzuguren et al., 2023)
			(Jonker et al., 2023)
			(Shi et al., 2023)
			(Şevik, 2024)
			(Esmaeili et al., 2025)

Table 2 Non-infectious agents causing abortions

Group		Non-infectious agent	Citation
Maternal causes	Metabolic and nutritional causes	Pregnancy toxemia	(Lima et al., 2016)
			(Esmaeili et al., 2025)
		Vitamin E / selenium deficiency	(Moeller, 2001)
			(Chanton-Greutmann et al., 2002)
			(Esmaeili et al., 2025)
		Copper deficiency	(Moeller, 2001)
		Trace mineral deficiency	(Ndou et al., 2012) not available
	Toxic causes	Aflatoxin B_1	(Maryamma et al., 1990)
Fetal causes	Developmental abnormalities	Congenital malformations	(Moeller, 2001)
			(Chanton-Greutmann et al., 2002)
			(van den Brom et al., 2012)
			(Esmaeili et al., 2025)
		Fetal thyroid hyperplasia	(Moeller, 2001)
			(Szeredi et al., 2006)
			(Szeredi et al., 2020)
			(Esmaeili et al., 2025)
	Mechanical and traumatic causes	Umbilical cord torsion	(Sobanaasree et al., 2017)
		Uterine torsion	(Jakkali et al., 2022)
		Fetal trauma	(Esmaeili et al., 2025)
Iatrogenic and experimentally induced causes	Nutritional and dietary manipulations		(Hussain, Ropstad, & Andresen, 1996)
			(Hussain, Waldeland, et al., 1996)
			(Pamo et al., 2006)
			(Mellado et al., 2014)
			(McGregor, 2016)
			(Zainal Ulum et al., 2021)
			(Affan et al., 2022)
	Experimentally administered toxins	Amorimia septentrionalis	(Da Silva et al., 2017)
			(Lopes et al., 2019)
		Claviceps purpurea	(Vogt Engeland et al., 1998)
		Leucaena leucocephala	(Sastry & Singh, 2008)
		Mimosa tenuiflora	(Dantas et al., 2012)
		Poincianella pyramidalis	(J. R. d. Santos et al., 2018)
			(Santos Dos Reis, Suélen Dias et al., 2016)
		Stryphnodendron fissuratum	(Albuquerque et al., 2011)
		Tetrapterys multiglandulosa	(Melo et al., 2001)
	Medical interventions	$PGF_2\alpha$	(Yong et al., 2010)
			(Kawu et al., 2013)
			(Chen et al., 2016)
		Administration of selenium	(Amini et al., 2011)
		Vaccination	(Zundel et al., 1992)
			(Villa et al., 2008)
			(Kamal, 2009)
			(Esmaeili et al., 2024)

Table 3 Unknown agents of abortion

Unknown agents	Citation
	(Moeller, 2001)
	(Chanton-Greutmann et al., 2002)
	(Szeredi et al., 2006)
	(Navarro et al., 2009)
	(van den Brom et al., 2012)
	(Hazlett et al., 2013)
	(van Engelen et al., 2014)
	(Omidi, 2015)
	(Díaz-Cao et al., 2018)
	(Szeredi et al., 2020)
	(Esmaeili et al., 2025)
Suspicious for infection	(Moeller, 2001)
	(Chanton-Greutmann et al., 2002)
	(van den Brom et al., 2012)
	(van Engelen et al., 2014)
	(Szeredi et al., 2020)

References

Ababneh, H. S., Ababneh, M. M. K., Hananeh, W. M., Alsheyab, F. M., Jawasreh, K. I., Al-Gharaibeh, M. A., & Ababneh, M. M. (2014). Molecular identification of chlamydial cause of abortion in small ruminants in Jordan. *Tropical Animal Health and Production*, *46*(8), 1407–1412. https://doi.org/10.1007/s11250-014-0654-x

Abdel-Moein, K. A., & Hamza, D. A. (2017). The burden of Coxiella burnetii among aborted dairy animals in Egypt and its public health implications. *Acta Tropica*, *166*, 92–95. https://doi.org/10.1016/j.actatropica.2016.11.011

Abnaroodheleh, F., Emadi, A., & Dadar, M. (2021). Seroprevalence of brucellosis and chlamydiosis in sheep and goats with history of abortion in Iran. *Small Ruminant Research : The Journal of the International Goat Association*, *202*, 106459. https://doi.org/10.1016/j.smallrumres.2021.106459

Affan, A. A., Salleh, A., Zamri-Saad, M., Jayanegara, A., & Hassim, H. A. (2022). Clinical Signs and Blood Variables of Pregnancy Toxemia Goats during Late Gestation and Postpartum. *Tropical Animal Science Journal*, *45*(1), 84–90. https://doi.org/10.5398/tasj.2022.45.1.84

Akman, A., & Okur Gumusova, S. (2023). The border disease virus (BDV) prevalence and genetic typing in ruminant flocks in Turkey. *Veterinaria Italiana*, *59*(2). https://doi.org/10.12834/VetIt.2693.17780.2

Al-Ani, F. K., El-Qaderi, S., Hailat, N. Q., Razziq, R., & Al-Darraji, A. M. (2004). Human and animal brucellosis in Jordan between 1996 and 1998: A study. *Revue Scientifique Et Technique (International Office of Epizootics)*, *23*(3), 831–840. https://doi.org/10.20506/rst.23.3.1528

Albayrak, H., & Özan, E. (2012). The investigation of pestivirus and rift valley fever virus infections in aborted ruminant foetuses in the blacksea region in Turkey. *Kafkas Universitesi Veteriner Fakultesi Dergisi*, *18*(3), 457–461. https://doi.org/10.9775/kvfd.2011.5648

Albuquerque, R. F., Evêncio-Neto, J., Freitas, S. H., Dória, R. G., Saurini, N. O., Colodel, E. M., Riet-Correa, F., & Mendonça, F. S. (2011). Abortion in goats after experimental administration of Stryphnodendron fissuratum (Mimosoideae). *Toxicon*, *58*(6-7), 602–605. https://doi.org/10.1016/j.toxicon.2011.07.017

Aldomy, F., Jahans, K. L., & Altarazi, Y. H. (1992). Isolation of Brucella melitensis from aborting ruminants in Jordan. *Journal of Comparative Pathology*, *107*(2), 239–242. https://doi.org/10.1016/0021-9975(92)90040-2

Alirezaei, A., Khalili, M., Baseri, N., Esmaeili, S., Mohammadi Damaneh, E., & Kazeminia, S. (2024). Molecular detection of Brucella species among aborted small ruminants in southeast Iran. *Brazilian Journal of Microbiology*, *55*(1), 911–917. https://doi.org/10.1007/s42770-023-01191-z

Al-Majali, A. M. (2005). Seroepidemiology of caprine Brucellosis in Jordan. *Small Ruminant Research*, *58*(1), 13–18. https://doi.org/10.1016/j.smallrumres.2004.07.013

Al-Mufarrej, S. I., Hussein, M. F., Aljumaah, R. S., & ElNabi, A. R. G. (2011). Toxoplasmosis in goats in Riyadh, Saudi Arabia. *Journal of Animal and Veterinary Advances*, *10*(21), 2779–2782. https://doi.org/10.3923/javaa.2011.2779.2782

Álvarez-Alonso, R., Basterretxea, M., Barandika, J. F., Hurtado, A., Idiazabal, J., Jado, I., Beraza, X., Montes, M., Liendo, P., & García-Pérez, A. L. (2018). A Q fever outbreak with a high rate of abortions at a dairy goat farm: Coxiella burnetii shedding, environmental contamination, and viability. *Applied and Environmental Microbiology*, *84*(20). https://doi.org/10.1128/AEM.01650-18

Alzuguren, O., Domínguez, L., Chacón, G., Benito, A. A., & Mencía-Ares, O. (2023). Infectious abortions in small domestic ruminants in the Iberian Peninsula: Optimization of sampling procedures for molecular diagnostics. *Frontiers in Veterinary Science*, *10*, 1152289. https://doi.org/10.3389/fvets.2023.1152289

Amini, K., Simko, E., & Davies, J. L. (2011). Diagnostic exercise: Sudden death associated with myocardial contraction band necrosis in boer goat kids. *Veterinary Pathology*, *48*(6), 1212–1215. https://doi.org/10.1177/0300985810381246

Amouei, A., Sharif, M., Sarvi, S., Bagheri Nejad, R., Aghayan, S. A., Hashemi-Soteh, M. B., Mizani, A., Hosseini, S. A., Gholami, S., Sadeghi, A., Sarafrazi, M., & Daryani, A. (2019). Aetiology of livestock fetal mortality in Mazandaran province, Iran. *PeerJ*, *6*, e5920. https://doi.org/10.7717/peerj.5920

Astobiza, I., Tilburg, J., Piñero, A., Hurtado, A., García-Pérez, A. L., Nabuurs-Franssen, M. H., & Klaassen, C. (2012). Genotyping of Coxiella burnetii from domestic ruminants in northern Spain. *BMC Veterinary Research*, *8*. https://doi.org/10.1186/1746-6148-8-241

Atwa, E. I., & Rady, F. M. (2007). Bacteria and fungi associated with abortion in sheep and goat in Menoufiea Governorate. *Assiut Veterinary Medical Journal*, *53*(113), 1–23. https://doi.org/10.21608/avmj.2007.176310

Aymée, L., Di Azevedo, Maria Isabel Nogueira, de Melo, Juliana Dos Santos Loria, Balaro, M. F. A., Martins, Gabriel Mendes de Souza, Consalter, A., Da Leite, J. S., Carvalho-Costa, F. A., & Lilenbaum, W. (2022). Leptospira noguchii associated to reproductive disease in ruminants. *Transboundary and Emerging Diseases*, *69*(5), 3103–3108. https://doi.org/10.1111/tbed.14377

Bari, A., Yeasmin, F., & Alam, M. (1993). Pathology of naturally occurring toxoplasma abortion and neonatal mortality in Black Bengal goat. *Small Ruminant Research : The Journal of the International Goat Association*, *10*(4), 349–355. https://doi.org/10.1016/0921-4488(93)90139-9

Barr, B. C., Anderson, M. L., Woods, L. W., Dubey, J. P., & Conrad, P. A. (1992). Neospora-like protozoal infections associated with abortion in goats. *Journal of Veterinary Diagnostic Investigation : Official Publication of the American Association of*

Veterinary Laboratory Diagnosticians, Inc, *4*(3), 365–367. https://doi.org/10.1177/104063879200400331

Basso, W., Holenweger, F., Schares, G., Müller, N., Campero, L. M., Ardüser, F., Moore-Jones, G., Frey, C. F., & Zanolari, P. (2022). Toxoplasma gondii and Neospora caninum infections in sheep and goats in Switzerland: Seroprevalence and occurrence in aborted foetuses. *Food and Waterborne Parasitology*, *28*, e00176. https://doi.org/10.1016/j.fawpar.2022.e00176

Batista, J. S., dos Santos, W., Sousa, A. de, Da Silva Teófilo, T., Bezerra, A., Rodrigues, V., Da Silva Filho, J. A., Cavalcante, T. V., Freitas Mendonça Costa, K. M. de, & Viana, G. A. (2022). Abortion and congenital transmission of Trypanosoma vivax in goats and ewes in semiarid northeastern Brazil. *Research in Veterinary Science*, *149*, 125–127. https://doi.org/10.1016/j.rvsc.2022.06.009

Behroozikhah, A. M., Dadar, M., Asli, E., & Hosseini, S. D. (2022). Molecular survey of Brucella melitensis field isolates using sequence-based PCR of outer membrane protein 31. *Archives of Razi Institute*, *77*(3), 1261–1267. https://doi.org/10.22092/ARI.2021.355107.1664

Biobaku, K. T., Odetokun, I. A., Raji, L. O., Olurode, S. O., & Ameen, S. A. (2016). A case report of abortion induced by Aspergillus mycotoxicosis in Sokoto red goat. *Alexandria Journal of Veterinary Sciences*, *49*(1), 91–94. https://doi.org/10.5455/ajvs.202495

Borhani, R., Latifian, M., Khalili, M., Jajarmi, M., & Esmaeili, S. (2024). Molecular investigation of Coxiella burnetii in aborted fetus of small ruminants in southeast Iran. *Brazilian Journal of Microbiology*, *55*(1), 919–924. https://doi.org/10.1007/s42770-023-01202-z

Brijesh Bhardwaj, B. B., Rajesh Chahota, R. C., Shilpi Gupta, S. G., Priyanka Malik, P. M., & Mandeep Sharma, M. S. (2017). Identification of chlamydial strains causing abortions and pneumonia in sheep and goat flocks during trans Himalayan seasonal migration in the northern region of India. *Veterinarski Arhiv*, *87*(2), 157–170. http://www.vef.hr/vetarhiv

British Veterinary Association (2018). Chronic copper toxicity in Boer goats. *The Veterinary Record*, *182*(20), 566–569. https://doi.org/10.1136/vr.k2188

Broaddus, C. C., Lamm, C. G., Kapil, S., Dawson, L., & Holyoak, G. R. (2009). Bovine viral diarrhea virus abortion in goats housed with persistently infected cattle. *Veterinary Pathology*, *46*(1), 45–53. https://doi.org/10.1354/vp.46-1-45

Büyükcangaz, E., Sen, A., & Kahya, S. (2009). Isolation and biotyping of Brucella melitensis from aborted sheep and goat fetuses. *Turkish Journal of Veterinary & Animal Sciences*, *33*(4), 311–316. https://doi.org/10.3906/vet-0802-30

Cagirgan, A. A., Pekmez, K., Kaplan, M., Arslan, F., & Kalayci, G. (2022). Molecular investigation of most important viruses causing abortion in small ruminants: The importance of akabane virus. *Revista MVZ Cordoba*, *27*(1). https://doi.org/10.21897/rmvz.2356

Caldeira, F., Ubiali, D. G., Godoy, I. de, Dutra, V., Aguiar, D. M. de, Melo, A., Riet-Correa, F., Colodel, E. M., & Pescador, C. A. (2011). Outbreak of caprine abortion by Toxoplasma gondii in Midwest Brazil. *Pesquisa Veterinaria Brasileira*, *31*(11), 933–937. https://doi.org/10.1590/S0100-736X2011001100001

Campero, L. M., Gos, M. L., Moore, D. P., Regidor-Cerrillo, J., Unzaga, J. M., Moré, G., Ortega-Mora, L. M., & Venturini, M. C. (2018). Microsatellite pattern analysis of Neospora caninum from a naturally infected goat fetus. *Veterinary Parasitology*, *255*, 58–60. https://doi.org/10.1016/j.vetpar.2018.03.024

Campos-Hernández, E., Vázquez-Chagoyán, J. C., Salem, A. Z. M., Saltijeral-Oaxaca, J. A., Escalante-Ochoa, C., López-Heydeck, S. M., & Oca-Jiménez, R. M. de (2014). Prevalence and molecular identification of Chlamydia abortus in commercial dairy goat farms in a hot region in Mexico. *Tropical Animal Health and Production*, *46*(6), 919–924. https://doi.org/10.1007/s11250-014-0585-6

Cantas, L., Muwonge, A., Sareyyupoglu, B., Yardimci, H., & Skjerve, E. (2011). Q fever abortions in ruminants and associated on-farm risk factors in northern Cyprus. *BMC Veterinary Research*, *7*(1). https://doi.org/10.1186/1746-6148-7-13

Chanton-Greutmann, H., Thoma, R., Corboz, L., Borel, N., & Pospischil, A. (2002). Abortion in small ruminants in Switzerland: Investigations during two lambing seasons with special regard to Chlamydiae. *Schweizer Archiv Fur Tierheilkunde*, *144*(9), 483–492. https://doi.org/10.1024/0036-7281.144.9.483

Chauhan, H. C., Biswas, S. K., Chand, K., Rehman, W., Das, B., Dadawala, A. I., Chandel, B. S., Kher, H. N., & Mondal, B. (2014). Isolation of bluetongue virus serotype 1 from aborted goat fetuses. *Revue Scientifique Et Technique (International Office of Epizootics)*, *33*(3), 803–812. https://doi.org/10.20506/rst.33.3.2319

Chen, Y., Lv, W., Jia, J., Wang, J., & Yang, J. (2016). Evaluation of serum concentrations of tumor necrosis factor (TNF)-α, interleukin (IL)-2, IL-10, and nitric oxide (NO) during the estrous cycle, early pregnancy and abortion in goats. *Animal Reproduction Science*, *174*, 73–79. https://doi.org/10.1016/j.anireprosci.2016.09.007

Chénier, S., Montpetit, C., & Hélie, P. (2004). Caprine herpesvirus- 1 abortion storm in a goat herd in Quebec. *The Canadian Veterinary Journal = La Revue Veterinaire Canadienne*, *45*(3), 241–243.

Chisu, V., Loi, F., Mura, L., Tanda, A., Chessa, G., & Masala, G. (2021). Molecular detection of Theileria sergentii/orientalis/buffeli and Ehrlichia canis from aborted ovine and caprine products in Sardinia, Italy. *Veterinary Medicine and Science*, *7*(5), 1762–1768. https://doi.org/10.1002/vms3.510

Cho, S. H., Mete, A., Cueva, I., Macías-Rioseco, M., Fritz, H., Streitenberger, N., & Gonzales-Viera, O. (2025). Yersinia pseudotuberculosis and Y. Enterocolitica abortions in sheep and goats in California: A series of cases diagnosed at CAHFS

laboratories, 2002-2023. *Journal of Veterinary Diagnostic Investigation.* Advance online publication. https://doi.org/10.1177/10406387251324883

Chochlakis, D., Giadinis, N. D., Petridou, E. J., Filioussis, G., Tselentis, Y., Psaroulaki, A., Ioannidou, E., Papanikolopoulou, V., & Karatzias, H. (2020). Molecular evidence of Anaplasma phagocytophilumin aborted goat fetuses and placenta. *Veterinaria Italiana*, *56*(4), 302–303. https://doi.org/10.12834/VetIt.1173.6516.2

Coe, S. E., Dalton, M. F., Anis, E., Wilkes, R. P., & Howerth, E. W. (2020). Pathology in practice. *Journal of the American Veterinary Medical Association*, *256*(2), 183–186. https://doi.org/10.2460/javma.256.2.183

Copeland, S., Chirino-Trejo, M., Bourque, P., & Biernacki, A. (1991). Saskatchewan. Abortion due to Coxiella burnetii (Q fever) in a goat. *The Canadian Veterinary Journal = La Revue Veterinaire Canadienne*, *32*(4), 245.

Costa, R. C., Orlando, D. R., Abreu, C. C., Nakagaki, K. Y. R., Mesquita, L. P., Nascimento, L. C., Silva, A. C., Maiorka, P. C., Peconick, A. P., Raymundo, D. L., & Varaschin, M. S. (2014). Histological and immunohistochemical characterization of the inflammatory and glial cells in the central nervous system of goat fetuses and adult male goats naturally infected with Neospora caninum. *BMC Veterinary Research*, *10*, 291. https://doi.org/10.1186/s12917-014-0291-7

Da Silva, L., Pessoa, D., Lopes, J. R., Dos Santos, J., Olinda, R. G., & Riet-Correa, F. (2017). Embryonic death and abortion in goats caused by ingestion of Amorimia septentrionalis. *Pesquisa Veterinaria Brasileira*, *37*(12), 1401–1404. https://doi.org/10.1590/S0100-736X2017001200007

Dadar, M., & Alamian, S. (2021a). Identification of main Brucella species implicated in ovine and caprine abortion cases by molecular and classical methods. *Archives of Razi Institute*, *76*(1), 51–60. https://doi.org/10.22092/ari.2019.128003.1398

Dadar, M., & Alamian, S. (2021b). Investigation of small ruminant brucellosis among smallholder farms: The missing link in control programmes of endemic areas. *Zoonoses and Public Health*, *68*(5), 376–383. https://doi.org/10.1111/zph.12796

Dadar, M., & Alamian, S. (2025). In silico MLVA analysis of Brucella melitensis from human and livestock in Iran. *Current Microbiology*, *82*(2), 74. https://doi.org/10.1007/s00284-024-03940-1

Dadar, M., Alamian, S., Tadayon, K., Ashford, R. T., & Whatmore, A. M. (2022). Molecular characterization of zoonotic Brucella species isolated from animal and human samples in Iran. *Acta Tropica*, *229*, 106363. https://doi.org/10.1016/j.actatropica.2022.106363

Dadar, M., Brangsch, H., Alamian, S., Neubauer, H., & Wareth, G. (2023). Whole-genome sequencing for genetic diversity analysis of Iranian Brucella spp. Isolated from humans and livestock. *One Health*, *16*. https://doi.org/10.1016/j.onehlt.2023.100483

Dantas, A. F. M., Riet-Correa, F., Medeiros, R. M. T., Lopes, J. R., Gardner, D. R., Panter, K., & Mota, R. A. (2012). Embryonic death in goats caused by the ingestion

of Mimosa tenuiflora. *Toxicon*, *59*(5), 555–557. https://doi.org/10.1016/j.toxicon.2011.11.020

de Oliveira Junior, Ivam Moreira, Mesquita, Lucas Emanuel Dos Santos, Miranda, D. N. P., Gomes, T. A., Vasconcelos, B. K. S., Penha, L. C., Silveira, L. C. S., Redondo, A. R. R., Costa, R. C., Bruhn, F. R. P., Raymundo, D. L., Wouters, A. T. B., Wouters, F., & Varaschin, M. S. (2020). Endogenous transplacental transmission of Neospora caninum in successive generations of congenitally infected goats. *Veterinary Parasitology*, *284*, 109191. https://doi.org/10.1016/j.vetpar.2020.109191

de Oliveira, Júnior Mário Baltazar, Almeida, J. C. de, de Melo, Renata Pimentel Bandeira, Barros, L. D. de, Garcia, J. L., Andrade, M. R., Porto, W. J. N., Regidor-Cerrillo, J., Ortega-Mora, L. M., Oliveira, Andréa Alice da Fonseca, & Mota, R. A. (2018). First description of clonal lineage type II (genotype #1) of Toxoplasma gondii in abortion outbreak in goats. *Experimental Parasitology*, *188*, 21–25. https://doi.org/10.1016/j.exppara.2018.03.008

de Oliveira, Júnior Mário Baltazar, Rozental, T., de Lemos, Elba Regina Sampaio, Forneas, D., Ortega-Mora, L. M., Porto, W. J. N., da Fonseca Oliveira, Andréa Alice, & Mota, R. A. (2018). Coxiella burnetii in dairy goats with a history of reproductive disorders in Brazil. *Acta Tropica*, *183*, 19–22. https://doi.org/10.1016/j.actatropica.2018.04.010

Demirpence, M., Saytekin, A. M., Sareyyupoglu, B., & Esendal, O. M. (2022). Isolation and characterisation of Brucella melitensis by bacteriological and molecular methods from livestock in North Cyprus. *Veterinarni Medicina*, *67*(10), 497–509. https://doi.org/10.17221/150/2021-VETMED

Di Blasio, A., Traversa, A., Giacometti, F., Chiesa, F., Piva, S., Decastelli, L., Dondo, A., Gallina, S., & Zoppi, S. (2019). Isolation of Arcobacter species and other neglected opportunistic agents from aborted bovine and caprine fetuses. *BMC Veterinary Research*, *15*(1), 257. https://doi.org/10.1186/s12917-019-2009-3

Di Paolo, L. A., Alvarado Pinedo, M. F., Origlia, J., Fernández, G., Uzal, F. A., & Travería, G. E. (2019). First report of caprine abortions due to Chlamydia abortus in Argentina. *Veterinary Medicine and Science*, *5*(2), 162–167. https://doi.org/10.1002/vms3.145

Díaz-Cao, J. M., Prieto, A., López-Lorenzo, G., Díaz-Fernández, P., López-Sández, C., Morrondo, P., & Fernández-Rodríguez, G. (2018). Broadening the diagnosis panel of reproductive pathogens associated with abortion in ruminants. *Spanish Journal of Agricultural Research*, *16*(2). https://doi.org/10.5424/sjar/2018162-12180

Dominguez, M., Gache, K., Touratier, A., Perrin, J.-B., Fediaevsky, A., Collin, E., Bréard, E., Sailleau, C., Viarouge, C., Zanella, G., Zientara, S., Hendrikx, P., & Calavas, D. (2014). Spread and impact of the Schmallenberg virus epidemic in France in 2012-2013. *BMC Veterinary Research*, *10*(1). https://doi.org/10.1186/s12917-014-0248-x

Dubey, J. P., Morales, J. A., Villalobos, P., Lindsay, D. S., Blagburn, B. L., & Topper, M. J. (1996). Neosporosis-associated abortion in a dairy goat. *Journal of the American Veterinary Medical Association*, *208*(2), 263–265. https://doi.org/10.2460/javma.1996.208.02.263

Edwards, J. F., Angulo, A. B., & Pannill, E. C. (2003). Theriogenology question of the month. *Journal of the American Veterinary Medical Association*, *222*(10), 1361–1362. https://doi.org/10.2460/javma.2003.222.1361

Eleni, C., Crotti, S., Manuali, E., Costarelli, S., Filippini, G., Moscati, L., & Magnino, S. (2004). Detection of Neospora caninum in an aborted goat foetus. *Veterinary Parasitology*, *123*(3-4), 271–274. https://doi.org/10.1016/j.vetpar.2004.06.017

Engeland, I. V., Waldeland, H., Andresen, Ø., Løken, T., Björkman, C., & Bjerkås, I. (1998). Foetal loss in dairy goats: An epidemiological study in 22 herds. *Small Ruminant Research*, *30*(1), 37–48. https://doi.org/10.1016/s0921-4488(98)00084-4

Escalante-Ochoa, C., Díaz-Aparicio, E., Segundo-Zaragoza, C., & Suárez-Güemes, F. (1997). Isolation of Chlamydia psittaci involved in abortion of goats in Mexico: First report. *Revista Latinoamericana De Microbiologia*, *39*(3-4), 117–121. https://www.scopus.com/inward/record.uri?eid=2-s2.0-0031545660&partnerID=40&md5=1ef23b9a6eefeefe84fc06e32901e25e

Esmaeili, H., Ghorani, M., Hamidiya, Z., Joghataei, S. M., Villanueva-Saz, S., & Lacasta, D. (2025). Causes of abortion in Iranian goat herds and associated risk factors. *Preventive Veterinary Medicine*, *234*, 106381. https://doi.org/10.1016/j.prevetmed.2024.106381

Esmaeili, H., Ghorani, M., Joghataei, S. M., Villanueva-Saz, S., & Lacasta, D. (2024). Live attenuated goatpox vaccination in pregnant Murcia-Granada goats: Dosage implications and outcomes. *BMC Veterinary Research*, *20*(1), 544. https://doi.org/10.1186/s12917-024-04395-z

Esmaeilnejad, B., Tavassoli, M., Samiei, A., & Hajipour, N. (2018). Molecular verification of transplacental transmission of Theileria lestoquardi in goat. *Parasitology Research*, *117*(10), 3315–3318. https://doi.org/10.1007/s00436-018-6007-3

Fayez, M., Elmoslemany, A., Alorabi, M., Alkafafy, M., Qasim, I., Al-Marri, T., & Elsohaby, I. (2021). Seroprevalence and risk factors associated with Chlamydia abortus infection in sheep and goats in eastern Saudi Arabia. *Pathogens*, *10*(4). https://doi.org/10.3390/pathogens10040489

Giadinis, N. D., Lafi, S. Q., Ioannidou, E., Papadopoulos, E., Terpsidis, K., Karanikolas, G., Petridou, E. J., Brozos, C., & Karatzias, H. (2013). Reduction of the abortion rate due to Toxoplasma in 3 goat herds following administration of sulfadimidine. *The Canadian Veterinary Journal = La Revue Veterinaire Canadienne*, *54*(11), 1080–1082.

Giannitti, F., Anderson, M. L., Miller, M., Rowe, J., Sverlow, K., Vasquez, M., & Cantón, G. (2016). Chlamydia pecorum: Fetal and placental lesions in sporadic caprine abortion. *Journal of Veterinary Diagnostic Investigation : Official Publication of the*

American Association of Veterinary Laboratory Diagnosticians, Inc, *28*(2), 184–189. https://doi.org/10.1177/1040638715625729

Giannitti, F., Barr, B. C., Brito, B. P., Uzal, F. A., Villanueva, M., & Anderson, M. L. (2014). Yersinia pseudotuberculosis infections in goats and other animals diagnosed at the California Animal Health and Food Safety Laboratory System: 1990-2012. *Journal of Veterinary Diagnostic Investigation*, *26*(1), 88–95. https://doi.org/10.1177/1040638713516624

Golender, N., Bumbarov, V., Eldar, A., Lorusso, A., Kenigswald, G., Varsano, J. S., David, D., Schainin, S., Dagoni, I., Gur, I., Kaplan, A., Gorohov, A., Koren, O., Oron, E., Khinich, Y., Sclamovich, I., Meir, A., & Savini, G. (2020). Bluetongue serotype 3 in Israel 2013-2018: Clinical manifestations of the disease and molecular characterization of Israeli strains. *Frontiers in Veterinary Science*, *7*, 112. https://doi.org/10.3389/fvets.2020.00112

Golender, N., Bumbarov, V., Kovtunenko, A., David, D., Guini-Rubinstein, M., Sol, A., Beer, M., Eldar, A., & Wernike, K. (2021). Identification and genetic characterization of viral pathogens in ruminant gestation abnormalities, Israel, 2015-2019. *Viruses*, *13*(11). https://doi.org/10.3390/v13112136

Golender, N., Varsano, J. S., Nissimyan, T., & Tiomkin, E. (2022). Identification of novel reassortant Shuni virus strain in clinical cases of Israeli ruminants, 2020-2021. *Tropical Medicine and Infectious Disease*, *7*(10). https://doi.org/10.3390/tropicalmed7100297

Gonzalez, J., Passantino, G., Esnal, A., Cuesta, N., García Vera, J. A., Mechelli, L., Saez, A., García Marín, J. F., & Tempesta, M. (2017). Abortion in goats by Caprine alphaherpesvirus 1 in Spain. *Reproduction in Domestic Animals = Zuchthygiene*, *52*(6), 1093–1096. https://doi.org/10.1111/rda.13034

Gufler, H., Grogger, R., Schmalzer, J., & Baumgartner, W. (1999). Toxoplasma-induced abortions in goats. *Wiener Tierarztliche Monatsschrift*, *86*(5), 155–159. https://www.scopus.com/inward/record.uri?eid=2-s2.0-0042731426&partnerID=40&md5=f34e94583d316148b370e4ce0a5502a2

Günaydin, E., Müştak, H. K., Sareyyüpoğlu, B., & Ata, Z. (2015). PCR detection of Coxiella burnetii in fetal abomasal contents of ruminants. *Kafkas Universitesi Veteriner Fakultesi Dergisi*, *21*(1), 69–73. https://doi.org/10.9775/kvfd.2014.11729

Hailat, N., Khlouf, S., Ababneh, M., & Brown, C. (2018). Pathological, immunohistochemical and molecular diagnosis of abortions in small ruminants in Jordan with Reference to Chlamydia abortus and Brucella melitensis. *Pakistan Veterinary Journal*, *38*(1), 109–112. https://doi.org/10.29261/pakvetj/2018.022

Hasan, T., Mannan, A., Hossain, D., Rekha, A., Hossan, M. M., Alim, M. A., & Uddin, A. M. (2021). Molecular detection of Toxoplasma gondii in aborted fetuses of goats in Chattogram, Bangladesh. *Veterinary World*, *14*(9), 2386–2391. https://doi.org/10.14202/vetworld.2021.2386-2391

Hazlett, M. J., McDowall, R., DeLay, J., Stalker, M., McEwen, B., van Dreumel, T., Spinato, M., Binnington, B., Slavic, D., Carman, S., & Cai, H. Y. (2013). A

prospective study of sheep and goat abortion using real-time polymerase chain reaction and cut point estimation shows Coxiella burnetii and Chlamydophila abortus infection concurrently with other major pathogens. *Journal of Veterinary Diagnostic Investigation : Official Publication of the American Association of Veterinary Laboratory Diagnosticians, Inc*, *25*(3), 359–368. https://doi.org/10.1177/1040638713484729

Heidari, S., Derakhshandeh, A., Firouzi, R., Ansari-Lari, M., Masoudian, M., & Eraghi, V. (2018). Molecular detection of Chlamydophila abortus, Coxiella burnetii, and Mycoplasma agalactiae in small ruminants' aborted fetuses in southern Iran. *Tropical Animal Health and Production*, *50*(4), 779–785. https://doi.org/10.1007/s11250-017-1494-2

Heinzelmann, M., Rodriguez-Campos, S., Kittl, S., Zanolari, P., & Hirsbrunner, G. (2020). Abortions and stillbirths caused by Coxiella burnetii in goats. *Schweizer Archiv fur Tierheilkunde*, *162*(10), 625–633. https://doi.org/10.17236/sat00275

Hemsley, C. M., Essex-Lopresti, A., Chisnall, T., Millar, M., Neale, S., Reichel, R., Norville, I. H., & Titball, R. W. (2023). MLVA and com1 genotyping of Coxiella burnetii in farmed ruminants in Great Britain. *Veterinary Microbiology*, *277*. https://doi.org/10.1016/j.vetmic.2022.109629

Herder, V., Wohlsein, P., Peters, M., Hansmann, F., & Baumgärtner, W. (2012). Salient lesions in domestic ruminants infected with the emerging so-called Schmallenberg virus in Germany. *Veterinary Pathology*, *49*(4), 588–591. https://doi.org/10.1177/0300985812447831

Hussain, Q., Ropstad, E., & Andresen, Ø. (1996). Effects of type and quality of roughage and energy level on plasma progesterone levels in pregnant goats. *Small Ruminant Research : The Journal of the International Goat Association*, *21*(2), 113–120. https://doi.org/10.1016/0921-4488(96)00867-X

Hussain, Q., Waldeland, H., Havrevoll, Ø., Eik, L. O., Andresen, Ø., & Engeland, I. V. (1996). Effect of type of roughage and energy level on reproductive performance of pregnant goats. *Small Ruminant Research : The Journal of the International Goat Association*, *21*(2), 97–103. https://doi.org/10.1016/0921-4488(96)00865-6

Irehan, B., Sonmez, A., Atalay, M. M., Ekinci, A. I., Celik, F., Durmus, N., Ciftci, A. T., & Simsek, S. (2022). Investigation of Toxoplasma gondii, Neospora caninum and Tritrichomonas foetus in abortions of cattle, sheep and goats in Turkey: Analysis by real-time PCR, conventional PCR and histopathological methods. *Comparative Immunology, Microbiology and Infectious Diseases*, *89*, 101867. https://doi.org/10.1016/j.cimid.2022.101867

Islam, M., Sultana, N., Mostaree, M., Sultana, S., Farjana, T., Pervin, M., & Khan, M. (2022). Detection of abortifacient infectious agents in clinical cases of Black Bengal goat. *Journal of Bangladesh Agricultural University*, *20*(0), 1. https://doi.org/10.5455/JBAU.105284

Ismael, A. B., Masoud, E. E., & El-Nabtity, S. M. (2009). Potential clinical role of propolis in treatment of clinical ovine and caprine listeriosis. *Veterinary Medical Journal (Giza)*, *57*(4), 723–736. https://doi.org/10.21608/vmjg.2011.368060

Jakkali, N. J., Doddagoudar, V., Bijurkar, R. G., Malashri, G., & Tandle, M. K. (2022). A novel, un-conventional detorsion method for correcting left-sided uterine torsion in goat. *Haryana Vet.*(61(SI)), 152–153.

Jones, R. M., Twomey, D. F., Hannon, S., Errington, J., Pritchard, G. C., & Sawyer, J. (2010). Detection of Coxiella burnetii in placenta and abortion samples from British ruminants using real-time PCR. *The Veterinary Record*, *167*(25), 965–967. https://doi.org/10.1136/vr.c4040

Jonker, A., Thompson, P. N., & Michel, A. L. (2023). Approaches to increase recovery of bacterial and fungal abortion agents in domestic ruminants. *Onderstepoort Journal of Veterinary Research*, *90*(1). https://doi.org/10.4102/ojvr.v90i1.2010

Kalender, H., Kiliç, A., Eröksüz, H., Muz, A., Kilinç, Ü., & Taşdemir, B. (2013). Identification of Chlamydophila abortus infection in aborting ewes and goats in Eastern Turkey. *Revue De Medecine Veterinaire*, *164*(6), 295–301. https://www.scopus.com/inward/record.uri?eid=2-s2.0-84878840013&partnerID=40&md5=6334061f6399bccae5798cfd919c6e57

Kamal, S. A. (2009). Pathological studies on postvaccinal reactions of Rift Valley fever in goats. *Virology Journal*, *6*. https://doi.org/10.1186/1743-422X-6-94

Karabasanavar, N., Madhavaprasad, C. B., Manjunatha, S. S., Bagalkote, P. S., Sajjan, S. A., & Sundareshan, S. (2016). Shipping fever outbreak in goats: An opportunistic association of Mannheimia haemolytica and Pasteurella multocida. *Indian Veterinary Journal*, *93*(6), 16–19. https://www.scopus.com/inward/record.uri?eid=2-s2.0-84989325847&partnerID=40&md5=6f811b977002b14944500aa88a6df2c5

Katsiolis, A., Papanikolaou, E., Stournara, A., Giakkoupi, P., Papadogiannakis, E., Zdragas, A., Giadinis, N. D., & Petridou, E. J. (2022). Molecular detection of Brucella spp. in ruminant herds in Greece. *Tropical Animal Health and Production*, *54*(3). https://doi.org/10.1007/s11250-022-03175-x

Kawu, M. U., Yaqub, L. S., Ayo, J. O., Rekwot, P. I., Habibu, B., Tauheed, M., Suleiman, M. M., Shittu, M., Aluwong, T., Isa, H. I., & Abdullahi, A. (2013). Abortion induction and post-abortion oestrous cycle pattern following administration of prostaglandin f2 alpha in Sokoto red goats. *Veterinary Research*, *6*(5), 105–108. https://www.scopus.com/inward/record.uri?eid=2-s2.0-84894075278&partnerID=40&md5=32e0f793202ed9842a6be8b7db096665

Kreizinger, Z., Szeredi, L., Bacsadi, Á., Nemes, C., Sugár, L., Varga, T., Sulyok, K. M., Szigeti, A., Ács, K., Tóbiás, E., Borel, N., & Gyuranecz, M. (2015). Occurrence of Coxiella burnetii and Chlamydiales species in abortions of domestic ruminants and in wild ruminants in Hungary, Central Europe. *Journal of Veterinary Diagnostic Investigation : Official Publication of the American Association of*

Veterinary Laboratory Diagnosticians, Inc, *27*(2), 206–210. https://doi.org/10.1177/1040638714563566

Lamm, C. G., Broaddus, C. C., & Holyoak, G. R. (2009). Distribution of bovine viral diarrhea virus antigen in aborted fetal and neonatal goats by immunohistochemistry. *Veterinary Pathology*, *46*(1), 54–58. https://doi.org/10.1354/vp.46-1-54

Liao, Y. K., Chain, C. Y., Lu, Y. S., Li, N. J., Tsai, H. J., & Liou, P. P. (1997). Epizootic of Chlamydia psittaci infection in goats in Taiwan. *Journal of Basic Microbiology*, *37*(5), 327–333. https://doi.org/10.1002/jobm.3620370505

Lima, M. S., Silveira, J. M., Carolino, N., Lamas, L. P., Pascoal, R. A., & Hjerpe, C. A. (2016). Usefulness of clinical observations and blood chemistry values for predicting clinical outcomes in dairy goats with pregnancy toxaemia. *Irish Veterinary Journal*, *69*. https://doi.org/10.1186/s13620-016-0075-4

Liu, P., Wu, J. Y., Ma, W. M., Yang, Y. M., Lv, L., Cai, J., Liu, Z. J., He, J. J., Shang, Y. J., Li, Z. C., & Cao, X. A. (2024). Molecular detection and characterization of Coxiella burnetii in aborted samples of livestock in China. *Acta Tropica*, *254*. https://doi.org/10.1016/j.actatropica.2024.107163

Lopes, J. R., Araújo, J., Pessoa, D., Lee, S., Cook, D., Riet-Correa, F., & Medeiros, R. (2019). Neonatal mortality associated with sodium monofluoracetate in kids fed with colostrum from goats ingesting Amorimia septentrionalis. *Pesquisa Veterinaria Brasileira*, *39*(3), 163–167. https://doi.org/10.1590/1678-5150-PVB-5949

Mackie, J. T., & Dubey, J. P. (1996). Congenital Sarcocystosis in a Saanen Goat. *The Journal of Parasitology*, *82*(2), 350. https://doi.org/10.2307/3284179

Mackie, J. T., Rahaley, R. S., & Nugent, R. (1992). Suspected Sarcocystis encephalitis in a stillborn kid. *Australian Veterinary Journal*, *69*(5), 114–115. https://doi.org/10.1111/j.1751-0813.1992.tb07466.x

Magouras, I., Hunninghaus, J., Scherrer, S., Wittenbrink, M. M., Hamburger, A., Stärk, K. D. C., & Schüpbach-Regula, G. (2017). Coxiella burnetii Infections in Small Ruminants and Humans in Switzerland. *Transboundary and Emerging Diseases*, *64*(1), 204–212. https://doi.org/10.1111/tbed.12362

Maksimović, Z., Jamaković, A., Semren, O., & Rifatbegović, M. (2022). Molecular detection of Brucella spp. in clinical samples of seropositive ruminants in Bosnia and Herzegovina. *Comparative Immunology, Microbiology and Infectious Diseases*, *86*. https://doi.org/10.1016/j.cimid.2022.101821

Malal, M. E., & Turkyilmaz, S. (2021). Identification and genotyping of Chlamydia abortus with MLVA from ruminant abortions in the Marmara region of Turkey. *Thai Journal of Veterinary Medicine*, *51*(1), 169–175. https://doi.org/10.14456/tjvm.2021.22

Mangena, M. L., Gcebe, N., Thompson, P. N., & Adesiyun, A. A. (2023). Q fever and toxoplasmosis in South African livestock and wildlife: a retrospective study on seropositivity, sporadic abortion, and stillbirth cases in livestock caused by

Coxiella burnetii. *BMC Veterinary Research*, *19*(1). https://doi.org/10.1186/s12917-023-03645-w

Maryamma, K. I., Manomohan, C. B., Valsala, K. V., Ramachandran, K. M., & Rajan, A. (1990). Investigations on a spontaneous outbreak of aflatoxin-induced abortions and neonatal deaths in goats. *Journal of Veterinary and Animal Sciences*, *21*(2), 84–86. https://ovidsp.ovid.com/ovidweb.cgi?T=JS&CSC=Y&NEWS=N&PAGE=fulltext&D=caba3&AN=19931214986

Masala, G., Porcu, R., Daga, C., Denti, S., Canu, G., Patta, C., & Tola, S. (2007). Detection of pathogens in ovine and caprine abortion samples from Sardinia, Italy, by PCR. *Journal of Veterinary Diagnostic Investigation*, *19*(1), 96–98. https://doi.org/10.1177/104063870701900116

Masala, G., Porcu, R., Madau, L., Tanda, A., Ibba, B., Satta, G., & Tola, S. (2003). Survey of ovine and caprine toxoplasmosis by IFAT and PCR assays in Sardinia, Italy. *Veterinary Parasitology*, *117*(1-2), 15–21. https://doi.org/10.1016/j.vetpar.2003.07.012

Masala, G., Porcu, R., Sanna, G., Chessa, G., Cillara, G., Chisu, V., & Tola, S. (2004). Occurrence, distribution, and role in abortion of Coxiella burnetii in sheep and goats in Sardinia, Italy. *Veterinary Microbiology*, *99*(3-4), 301–305. https://doi.org/10.1016/j.vetmic.2004.01.006

Masala, G., Porcu, R., Sanna, G., Tanda, A., & Tola, S. (2005). Role of Chlamydophila abortus in ovine and caprine abortion in Sardinia, Italy. *Veterinary Research Communications*, *29 Suppl 1*, 117–123. https://doi.org/10.1007/s11259-005-0842-2

McCoy, M. H., Montgomery, D. L., Bratanich, A. C., Cavender, J., Scharko, P. B., & Vickers, M. L. (2007). Serologic and reproductive findings after a herpesvirus-1 abortion storm in goats. *Journal of the American Veterinary Medical Association*, *231*(8), 1236–1239. https://doi.org/10.2460/javma.231.8.1236

McGregor, B. A. (2016). The effects of nutrition and parity on the development and productivity of Angora goats: 1. Manipulation of mid pregnancy nutrition on energy intake and maintenance requirement, kid birth weight, kid survival, doe live weight and mohair production. *Small Ruminant Research*, *145*, 65–75. https://doi.org/10.1016/j.smallrumres.2016.10.027

Mellado, M., Gaytán, L., Rodríguez, A., Macías-Cruz, U., Avendaño-Reyes, L., & García, J. E. (2014). Nutritive content of aborted and non-aborted goat diets on rangeland. *Veterinarija Ir Zootechnika*, *67*(89), 68–74. https://www.scopus.com/inward/record.uri?eid=2-s2.0-84907549458&partnerID=40&md5=2c99c14475ace619be867bb77cf44836

Melo, M. M., Vasconcelos, A. C., Dantas, G. C., Serakides, R., & Alzamora Filho, F. (2001). Experimental intoxication of pregnant goats with Tetrapterys multiglandulosa A. Juss. (Malpighiaceae). *Arquivo Brasileiro De Medicina*

Veterinaria E Zootecnia, *53*(1), 58–65. https://doi.org/10.1590/S0102-09352001000100009
Mesquita, L. P., Costa, R. C., Nogueira, C. I., Abreu, C. C., Orlando, D. R., Ascari Junior, I., Peconick, A. P., & Varaschin, M. S. (2018). Placental lesions associated with abortion and stillbirth in goats naturally infected by Neospora caninum 1. *Pesquisa Veterinaria Brasileira*, *38*(3), 444–449. https://doi.org/10.1590/1678-5150-PVB-4598
Mesquita, L. P., Nogueira, C. I., Costa, R. C., Orlando, D. R., Bruhn, F., Lopes, P., Nakagaki, K., Peconick, A. P., Seixas, J. N., Júnior, P., Raymundo, D. L., & Varaschin, M. S. (2013). Antibody kinetics in goats and conceptuses naturally infected with Neospora caninum. *Veterinary Parasitology*, *196*(3-4), 327–333. https://doi.org/10.1016/j.vetpar.2013.03.002
Moeller, R. B., JR (2001). Causes of caprine abortion: Diagnostic assessment of 211 cases (1991-1998). *Journal of Veterinary Diagnostic Investigation : Official Publication of the American Association of Veterinary Laboratory Diagnosticians, Inc*, *13*(3), 265–270. https://doi.org/10.1177/104063870101300317
Mohabati Mobarez, A., Baseri, N., Khalili, M., Mostafavi, E., & Esmaeili, S. (2023). Genotyping and phylogenetic analysis of Coxiella burnetii in domestic ruminant and clinical samples in Iran: Insights into Q fever epidemiology. *Scientific Reports*, *13*(1). https://doi.org/10.1038/s41598-023-47920-0
Mohabati Mobarez, A., Khalili, M., Mostafavi, E., & Esmaeili, S. (2021). Molecular detection of Coxiella burnetii infection in aborted samples of domestic ruminants in Iran. *PloS One*, *16*(4), e0250116. https://doi.org/10.1371/journal.pone.0250116
Mohammed, R. R., Tavassoli, M., Sidiq, K. R., & Esmaeilnejad, B. (2023). Prevalence of Neospora caninum as an etiologic agent of animal abortion in Kurdistan Region of Iraq. *Polish Journal of Veterinary Sciences*, *26*(3), 349–357. https://doi.org/10.24425/pjvs.2023.145039
Moore, J. D., Barr, B. C., Daft, B. M., & O'connor, M. T. (1991). Pathology and diagnosis of Coxiella burnetii infection in a goat herd. *Veterinary Pathology*, *28*(1), 81–84. https://doi.org/10.1177/030098589102800112
Moreno, B., Collantes-Fernández, E., Villa, A., Navarro, A., Regidor-Cerrillo, J., & Ortega-Mora, L. M. (2012). Occurrence of Neospora caninum and Toxoplasma gondii infections in ovine and caprine abortions. *Veterinary Parasitology*, *187*(1-2), 312–318. https://doi.org/10.1016/j.vetpar.2011.12.034
Moshkelani, S., Javaheri-Koupaei, M., Fathpour, H., & Alirezaei, M. (2011). Detection of Brucella melitensis from aborted caprine fetuses in Iran. *Global Veterinaria*, *6*(5), 495–497. https://www.scopus.com/inward/record.uri?eid=2-s2.0-80051947988&partnerID=40&md5=4edc596dc2fb0f0ef5834bab75bfdd6d
Murat, Ş. (2024). Potential role of peste des petits ruminants virus in small ruminant abortions. *Veterinary Journal (London, England : 1997)*, *306*, 106185. https://doi.org/10.1016/j.tvjl.2024.106185

Navarro, J. A., Ortega, N., Buendia, A. J., Gallego, M. C., Martínez, C. M., Caro, M. R., Sánchez, J., & Salinas, J. (2009). Diagnosis of placental pathogens in small ruminants by immunohistochemistry and PCR on paraffin-embedded samples. *Veterinary Record*, *165*(6), 175–178. https://doi.org/10.1136/vr.165.6.175

Ndou, R. V., Dlamini, M. L., Dara, O. B., Dzoma, B. M., Nyirenda, M., Le Motsei, & Bakunzi, F. R. (2012). Trace mineral deficiency in goats with history of abortions in Vhembe District of Limpopo Province, South Africa. *Asia Life Sciences*, 29–34.

Ntivuguruzwa, J. B., Kolo, F. B., Mwikarago, E. I., & van Heerden, H. (2022). Characterization of Brucella spp. And other abortigenic pathogens from aborted tissues of cattle and goats in Rwanda. *Veterinary Medicine and Science*, *8*(4), 1655–1663. https://doi.org/10.1002/vms3.805

Nunes, A., Yamasaki, E. M., Kim, P., Melo, R., Ribeiro-Andrade, M., Porto, W., & Mota, R. A. (2017). Transplacental transmission of Neospora caninum in naturally infected small ruminants from northeastern Brazil. *Pesquisa Veterinaria Brasileira*, *37*(9), 921–925. https://doi.org/10.1590/s0100-736x2017000900004

Oliveira, J. de, Silva, B. P., Ribeiro-Andrade, M., Porto, W., Melo, R. de, Junior, J., Da Fonseca Oliveira, A. A., & Mota, R. A. (2022). Toxoplasma gondii infection in goats: serological, pathological, and clinical monitoring during gestation. *Parasitology Research*, *121*(11), 3147–3153. https://doi.org/10.1007/s00436-022-07633-1

Omidi, A. (2015). Etiologic evaluation of late term abortions or stillbirths in some small ruminant flocks of South Khorasan province, Iran. *Istanbul Universitesi Veteriner Fakultesi Dergisi*, *41*(2), 199–204. https://doi.org/10.16988/iuvfd.2015.57738

Ozgen, E. K., Kilicoglu, Y., Yanmaz, B., Ozmen, M., Ulucan, M., Serifoglu Bagatir, P., Karadeniz Putur, E., Ormanci, S., Okumus, B., Iba Yilmaz, S., Karasahin, O., Aslan, M. H., Ozturk, M., Birinci, A., Bilgin, K., Tanriverdi Cayci, Y., & Tanyel, E. (2022). Molecular epidemiology of Coxiella burnetii detected in humans and domestic ruminants in Turkey. *Veterinary Microbiology*, *273*, 109519. https://doi.org/10.1016/j.vetmic.2022.109519

Palanivel, K. M., Sureshkumar, K., Sakthivelan, S. M., Kumarasamy, P., & Sivaselvam, S. N. (2012). Reproductive losses in boer x local goats caused by Brucella melitensis. *Indian Veterinary Journal*, *89*(11), 20–23. https://www.scopus.com/inward/record.uri?eid=2-s2.0-84869029473&partnerID=40&md5=313656e61416847c698c02a24682aa06

Pamo, E. T., Tendonkeng, F., Kana, J. R., Boukila, B., & Nanda, A. S. (2006). Effects of Calliandra calothyrsus and Leucaena leucocephala supplementary feeding on goat production in Cameroon. *Small Ruminant Research*, *65*(1-2), 31–37. https://doi.org/10.1016/j.smallrumres.2005.05.023

Partoandazanpoor, A., Sadeghi-Dehkordi, Z., Ekradi, L., Khordadmehr, M., Rassouli, M., & Sazmand, A. (2020). Molecular diagnosis and pathological study of Toxoplasma gondii in aborted caprine and ovine fetuses in borderline of Iran-Iraq. *Acta Parasitologica*, *65*(1), 187–192. https://doi.org/10.2478/s11686-019-00147-4

Pereira, G. O., Pereira, A. H., Brito, M. D., Pescador, C. A., & Ubiali, D. G. (2021). Toxoplasma gondii induced abortions in a goat herd in Rio de Janeiro, Brazil. *Ciencia Rural*, *51*(4). https://doi.org/10.1590/0103-8478cr20200568

Pestil, Z., Sait, A., Sayı, O., Ozbaser, F. T., & Bulut, H. (2020). Molecular epidemiology of peste des petits ruminants cases associated with abortion in sheep and goat in Marmara Region of Turkey, 2018. *Pakistan Veterinary Journal*, *40*(4), 494–498. https://doi.org/10.29261/pakvetj/2020.042

Piva, S., Mariella, J., Cricca, M., Giacometti, F., Brunetti, B., Mondo, E., Castelli, L. de, Romano, A., Ferrero, I., Ambretti, S., Roccaro, M., Merialdi, G., Scagliarini, A., Serraino, A., & Peli, A. (2021). Epidemiologic case investigation on the zoonotic transmission of Staphylococcus aureus infection from goat to veterinarians. *Zoonoses and Public Health*, *68*(6), 684–690. https://doi.org/10.1111/zph.12836

Pritchard, G. C., Smith, R. P., Errington, J., Hannon, S., Jones, R. M., & Mearns, R. (2011). Prevalence of Coxiella burnetii in livestock abortion material using PCR. *Veterinary Record*, *169*(15), 391. https://doi.org/10.1136/vr.d4693

Rajagunalan, S., Gururaj, K., Lakshmikantan, U., Murugan, M., Ganesan, A., Sundar, A., Sureshkannan, S., Andani, D., & Pawaiya, R. S. (2019). Detection of the presence of Coxiella burnetii in a case of goat abortion: A first report from India. *Tropical Animal Health and Production*, *51*(4), 983–986. https://doi.org/10.1007/s11250-018-1756-7

Ramo, María de Los Angeles, Benito, A. A., Quílez, J., Monteagudo, L. V., Baselga, C., & Tejedor, M. T. (2022). Coxiella burnetii and co-infections with other major pathogens causing abortion in small ruminant flocks in the Iberian Peninsula. *Animals : An Open Access Journal from MDPI*, *12*(24). https://doi.org/10.3390/ani12243454

Regidor-Cerrillo, J., Horcajo, P., Ceglie, L., Schiavon, E., Ortega-Mora, L. M., & Natale, A. (2020). Genetic characterization of Neospora caninum from Northern Italian cattle reveals high diversity in European N. Caninum populations. *Parasitology Research*, *119*(4), 1353–1362. https://doi.org/10.1007/s00436-020-06642-2

Reichel, R., Mearns, R., Brunton, L., Jones, R., Horigan, M., Vipond, R., Vincent, G., & Evans, S. (2012). Description of a Coxiella burnetii abortion outbreak in a dairy goat herd, and associated serology, PCR and genotyping results. *Research in Veterinary Science*, *93*(3), 1217–1224. https://doi.org/10.1016/j.rvsc.2012.04.007

Ribeiro, L. M., Herr, S., Chaparro, F., & van der Vyver, F. H. (1990). The isolation and serology of Brucella melitensis in a flock of goats in central RSA. *Onderstepoort Journal of Veterinary Research*, *57*(2), 143–144. https://www.scopus.com/inward/record.uri?eid=2-s2.0-0025442938&partnerID=40&md5=88e18845e61a3661b6dda5e1ee9d1ca4

Rodríguez, J. L., los Monteras, A. de, Herráez, P., Poveda, J. B., & Fernández, A. (1995). Isolation of Mycoplasma mycoides, mycoides (LC variant), from two naturally aborted caprine fetuses. *Theriogenology*, *44*(7), 1003–1009. https://doi.org/10.1016/0093-691X(95)00287-I

Rosamilia, A., Grattarola, C., Caruso, C., Peletto, S., Gobbi, E., Tarello, V., Caroggio, P., Dondo, A., Masoero, L., & Acutis, P. L. (2014). Detection of border disease virus (BDV) genotype 3 in Italian goat herds. *Veterinary Journal (London, England : 1997)*, *199*(3), 446–450. https://doi.org/10.1016/j.tvjl.2013.12.006

Sah, R. P., Dey, A. R., Rahman, A. K. M. Anisur, Alam, M. Z., & Talukder, M. H. (2019). Molecular detection of Toxoplasma gondii from aborted fetuses of sheep, goats and cattle in Bangladesh. *Veterinary Parasitology, Regional Studies and Reports*, *18*, 100347. https://doi.org/10.1016/j.vprsr.2019.100347

Sahin, O., Fitzgerald, C., Stroika, S., Zhao, S., Sippy, R. J., Kwan, P., Plummer, P. J., Han, J., Yaeger, M. J., & Zhang, Q. (2012). Molecular evidence for zoonotic transmission of an emergent, highly pathogenic Campylobacter jejuni clone in the United States. *Journal of Clinical Microbiology*, *50*(3), 680–687. https://doi.org/10.1128/JCM.06167-11

Sakmanoğlu, A., Uslu, A., Sayın, Z., Gölen, G. S., İlban, A., Padron-Perez, B., Karyeyen, Y., Gök, A., Tekindal, M. A., & Erganis, O. (2021). A one-year descriptive epidemiology of zoonotic abortifacient pathogen bacteria in farm animals in Turkey. *Comparative Immunology, Microbiology and Infectious Diseases*, *77*, 101665. https://doi.org/10.1016/j.cimid.2021.101665

Saleh, M., El-Hady, A. M. M., A. Abdelkader, S., S. S. Salem, H., M. Mohammed, M., A. El Shafei, A., & El-Shafei, M. (2021). Sero-prevalence and molecular identification of Coxiella burnetii (Q fever) among human and animals in Egypt. *Egyptian Journal of Veterinary Sciences*, *52*(1), 51–59. https://doi.org/10.21608/ejvs.2021.95033.1291

Samadi, A., Ababneh, M. M. K., Giadinis, N. D., & Lafi, S. Q. (2010). Ovine and caprine brucellosis (Brucella melitensis) in aborted animals in Jordanian sheep and goat flocks. *Veterinary Medicine International*, *2010*, 458695. https://doi.org/10.4061/2010/458695

Sanford, S. E., Josephson, G. K., & MacDonald, A. (1993). Ontario. Q fever abortions in a goat herd. *The Canadian Veterinary Journal = La Revue Veterinaire Canadienne*, *34*(4), 246. https://ovidsp.ovid.com/ovidweb.cgi?T=JS&CSC=Y&NEWS=N&PAGE=fulltext&D=caba3&AN=19932287454

Sanford, S. E., Josephson, G. K., & MacDonald, A. (1994). Coxiella burnetii (Q fever) abortion storms in goat herds after attendance at an annual fair. *The Canadian Veterinary Journal = La Revue Veterinaire Canadienne*, *35*(6), 376–378.

Sanjay Ghodasara, S. G., Ashish Roy, A. R., Rank, D. N., & Bhanderi, B. B. (2010). Identification of Brucella spp. From animals with reproductive disorders by polymerase chain reaction assay. *Buffalo Bulletin*, *29*(2), 98–108. http://ibic.lib.ku.ac.th/e-Bulletin/29-2.pdf

Santana, L. F., Rossi, G., Gaspar, R. C., Pinto, V., Oliveira, G., & Costa, A. (2013). Evidence of sexual transmission of Toxoplasma gondii in goats. *Small Ruminant Research*, *115*(1-3), 130–133. https://doi.org/10.1016/j.smallrumres.2013.08.008

Santos, J. R. d., Lopes, J. R., Medeiros, M. A., Campos, É. M., Medeiros, R. M., & Riet-Correa, F. (2018). Embryonic mortality and abortion in goats caused by ingestion of Poincianella pyramidalis. *Pesquisa Veterinária Brasileira*, *38*(7), 1259–1263. https://doi.org/10.1590/1678-5150-PVB-5480

Santos, S., Azenha, D., Oliveira, C., & Almeida, A. (2022). Coxiella burnetii and Chlamydia spp. coinfection in small ruminant abortion in Portugal. *Small Ruminant Research*, *207*. https://doi.org/10.1016/j.smallrumres.2022.106616

Santos Dos Reis, Suélen Dias, Oliveira, R. S. de, Correia Marcelino, S. A., Silva Almeida E Macêdo, Juliana Targino, Riet-Correa, F., Da Anunciação Pimentel, L., & Ocampos Pedroso, P. M. (2016). Congenital malformations and other reproductive losses in goats due to poisoning by Poincianella pyramidalis (Tul.) L.P. Queiroz (=Caesalpinia pyramidalis Tul.). *Toxicon*, *118*, 91–94. https://doi.org/10.1016/j.toxicon.2016.04.043

Sastry, M. S., & Singh, R. (2008). Toxic effects of subabul (Leucaena Leucocephala) on the thyroid and reproduction of female goats. *Indian Journal of Animal Sciences*, *78*(3), 251–253. https://www.scopus.com/inward/record.uri?eid=2-s2.0-48049092065&partnerID=40&md5=2d82513e01004329f9dfaffe42a6108d

Savini, G., Lorusso, A., Paladini, C., Migliaccio, P., Di Gennaro, A., Di Provvido, A., Scacchia, M., & Monaco, F. (2014). Bluetongue serotype 2 and 9 modified live vaccine viruses as causative agents of abortion in livestock: A retrospective analysis in Italy. *Transboundary and Emerging Diseases*, *61*(1), 69–74. https://doi.org/10.1111/tbed.12004

Scarcelli, E., Piatti, R. M., Harakava, R., Miyashiro, S., Campos, F. R., Souza, M., Cardoso, M. V., Teixeira, S. R., & Genovez, M. E. (2009). Use of pcr-rflp of thefla a gene for detection and subtyping of Campylobacter jejuni strains Potentially related to Guillain-barré syndrome, isolated from humans and animals. *Brazilian Journal of Microbiology*, *40*(4), 952–959. https://doi.org/10.1590/S1517-83822009000400029

Schnydrig, P., Overesch, G., Regli, W., Bee, A., & Rodriguez-Campos, S. (2018). Salmonella enterica subspecies diarizonae serovar 61:(k):1,5,(7) as cause of caprine abortion. *Small Ruminant Research*, *166*, 78–82. https://doi.org/10.1016/j.smallrumres.2018.07.022

Schnydrig, P., Vidal, S., Brodard, I., Frey, C. F., Posthaus, H., Perreten, V., & Rodriguez-Campos, S. (2017). Bacterial, fungal, parasitological and pathological analyses of abortions in small ruminants from 2012-2016. *Schweizer Archiv Fur Tierheilkunde*, *159*(12), 647–656. https://doi.org/10.17236/sat00136

Schöpf, K., Khaschabi, D., & Dackau, T. (1991). Abortusenzootie in einer Ziegenherde, bedingt durch Mischinfektion mit Coxiella burnetii und Chlamydia psittaci. Fallbericht. *Tierärztliche Praxis*, *19*(6), 630–634. https://www.embase.com/search/results?subaction=viewrecord&id=L22901164&from=export

Şevik, M. (2021). Genomic characterization of pestiviruses isolated from bovine, ovine and caprine foetuses in Turkey: A potentially new genotype of Pestivirus I species. *Transboundary and Emerging Diseases*, *68*(2), 417–426. https://doi.org/10.1111/tbed.13691

Şevik, M. (2024). A six-year epidemiological study of selected zoonotic abortifacient agents in ovine and caprine foetuses in Türkiye. *Epidemiology and Infection*, *152*, e173. https://doi.org/10.1017/S0950268824001699

Shahiduzzaman, M., Biswas, P., Kabir, A., Beni Amin, Abu Rakib M, Parijat, S. S., Ahmed, N., Hossain, M. Z., & Wakid, M. H. (2024). First report of Neospora caninum from aborted fetuses of cattle, sheep, and goats in Bangladesh. *Journal of Advanced Veterinary and Animal Research*, *11*(3), 618–626. https://doi.org/10.5455/javar.2024.k811

Sharawi, S. S. A., Yousef, M. R., Dokhan, K. Z., & Al-Hofufy, A. N. (2010). Virologic, serologic and pathologic studies into border disease virus (BDV) infection in a goat herd in Saudi Arabia. *Assiut Veterinary Medical Journal*, *56*(127), 1–12. https://doi.org/10.21608/avmj.2010.174238

Sharma, K. K., Vihol, P. D., Kalyani, I. H., Patel, D. R., & Rathod, P. H. (2017). Leptospira induced abortion in goats in Southern Gujarat. *Indian Journal of Veterinary Pathology*, *41*(4), 300–302. https://doi.org/10.5958/0973-970X.2017.00071.2

Sharma, S. P., Baipoledi, E. K., Nyange, J. F. C., & Tlagae, L. (2003). Isolation of Toxoplasma gondii from goats with history of reproductive disorders and the prevalence of Toxoplasma and chlamydial antibodies. *The Onderstepoort Journal of Veterinary Research*, *70*(1), 65–68. https://www.scopus.com/inward/record.uri?eid=2-s2.0-0041764460&partnerID=40&md5=7aa7781e89dc5c37da0153cfbe221eba

Shi, H., Hui, R., Zhou, M., Wang, L., Li, G., Bai, Y., & Yao, L. (2023). Abortion outbreak in pregnant goats and cows with coinfection of 'Candidatus Mycoplasma haemobos' and HoBi-like pestivirus. *Veterinary Microbiology*, *279*, 109690. https://doi.org/10.1016/j.vetmic.2023.109690

Silva Filho, M. de F., Erzinger, E., Da Cunha, I. A. L., Bugni, F. M., Hamada, F. N., Marana, E. R. M., Freire, R. L., Garcia, J. L., & Navarro, I. T. (2008). Toxoplasma gondii: Abortion outbreak in a goat herd from Southern Brazil. *Semina-Ciencias Agrarias*, *29*(4), 887–894. http://www.uel.br/proppg/portal/pages/arquivos/pesquisa/semina/pdf/semina_29_4_19_18.pdf

Singh, A., Gupta, V. K., Kumar, A., Singh, V. K., & Nayakwadi, S. (2013). 16s rRNA and omp31 gene based molecular characterization of field strains of B. Melitensis from aborted foetus of goats in India. *Scientific World Journal*, *2013*, 160376. https://doi.org/10.1155/2013/160376

Skinner, L. J., Timperley, A. C., Wightman, D., Chatterton, J. M., & Ho-Yen, D. O. (1990). Simultaneous diagnosis of toxoplasmosis in goats and goatowner's family.

Scandinavian Journal of Infectious Diseases, *22*(3), 359–361. https://doi.org/10.3109/00365549009027060

Sobanaasree, R., Raja, S., Vijayarajan, A., Prasath, N. B., Kumar, S. S., & Prabaharan, V. (2017). Fetal abortion and mummification due to umbilical cord torsion in a non descriptive goat. *Indian Journal of Animal Health*, *56*(2), 295–298. http://www.ijah.in

Špičić, S., Duvnjak, S., Zdelar-Tuk, M., Laroucau, K., Reil, I., Velić, L., Eterović, T., Pavlinec, Ž., Šegota, M., Habrun, B., & Cvetnić, Ž. (2019). Identification and MLVA genotyping of Chlamydia abortus from abortion cases in small ruminants in Croatia. *Veterinarska Stanica*, *50*(4), 307–314. https://www.scopus.com/inward/record.uri?eid=2-s2.0-85070885145&partnerID=40&md5=07a82561086aa928029cdab92659d9ab

Szeredi, L., & Bacsadi, Á. (2002). Detection of Chlamydophila (Chlamydia) abortus and Toxoplasma gondii in smears from cases of ovine and caprine abortion by the streptavidin-biotin method. *Journal of Comparative Pathology*, *127*(4), 257–263. https://doi.org/10.1053/jcpa.2002.0591

Szeredi, L., Dan, A., Malik, P., Janosi, S., & Hornyak, A. (2020). Low incidence of Schmallenberg virus infection in natural cases of abortion in domestic ruminants in Hungary. *Acta Veterinaria Hungarica*, *68*(1), 105–111. https://doi.org/10.1556/004.2020.00002

Szeredi, L., Jánosi, S., Tenk, M., Tekes, L., Bozsó, M., Deim, Z., & Molnár, T. (2006). Epidemiological and pathological study on the causes of abortion in sheep and goats in Hungary (1998-2005). *Acta Veterinaria Hungarica*, *54*(4), 503–515. https://doi.org/10.1556/AVet.54.2006.4.8

Toplu, N., Oğuzoğlu, T., Epikmen, E. T., & Aydoğan, A. (2011). Neuropathologic study of border disease virus in naturally infected fetal and neonatal small ruminants and its association with apoptosis. *Veterinary Pathology*, *48*(3), 576–583. https://doi.org/10.1177/0300985810371309

Tuncer-Göktuna, P., Alpay, G., Öner, E. B., & Yeşilbağ, K. (2016). The role of herpesviruses (BoHV-1 and BoHV-4) and pestiviruses (BVDV and BDV) in ruminant abortion cases in western Turkey. *Tropical Animal Health and Production*, *48*(5), 1021–1027. https://doi.org/10.1007/s11250-016-1050-5

Twomey, D. F., Errington, J., Hannon, S., Jones, R. M., Porter, T. A., Shock, A., & Pritchard, G. C. (2011). Q fever abortion outbreak in a British dairy goat herd. *Goat Veterinary Society Journal*, *27*, 73–80. http://www.goatvetsoc.co.uk

Unzaga, J. M., Moré, G., Bacigalupe, D., Rambeaud, M., Pardini, L., Dellarupe, A., Felice, L. de, Gos, M. L., & Venturini, M. C. (2014). Toxoplasma gondii and Neospora caninum infections in goat abortions from Argentina. *Parasitology International*, *63*(6), 865–867. https://doi.org/10.1016/j.parint.2014.07.009

Uzal, F. A., Woods, L., Stillian, M., Nordhausen, R., Read, D. H., van Kampen, H., Odani, J., Hietala, S., Hurley, E. J., Vickers, M. L., & Gard, S. M. (2004). Abortion and ulcerative posthitis associated with caprine herpesvirus-1 infection in goats

in California. *Journal of Veterinary Diagnostic Investigation : Official Publication of the American Association of Veterinary Laboratory Diagnosticians, Inc*, *16*(5), 478–484. https://doi.org/10.1177/104063870401600523

van Brom, R. den, Santman-Berends, I., Dijkman, R., Vellema, P., Dijkman, R., & van Engelen, E. (2021). An accessible diagnostic toolbox to detect bacterial causes of ovine and caprine abortion. *Pathogens*, *10*(9). https://doi.org/10.3390/pathogens10091147

van den Brom, R., Lievaart-Peterson, K., Luttikholt, S., Peperkamp, K., Wouda, W., & Vellema, P. (2012). Abortion in small ruminants in the Netherlands between 2006 and 2011. *Tijdschrift Voor Diergeneeskunde*, *137*(7), 450–457.

van der Walt, M., Rakaki, M. E., MacIntyre, C., Mendes, A., Junglen, S., Theron, C., Anthony, T., O'Dell, N., & Venter, M. (2023). Identification and molecular characterization of Shamonda virus in an aborted goat fetus in South Africa. *Pathogens*, *12*(9). https://doi.org/10.3390/pathogens12091100

van Engelen, E., Luttikholt, S., Peperkamp, K., Vellema, P., & van den Brom, R. (2014). Small ruminant abortions in the Netherlands during lambing season 2012-2013. *Veterinary Record*, *174*(20), 506. https://doi.org/10.1136/vr.102244

Vilela, V. L. R., Feitosa, T. F., Simões, S. V. D., Mota, R. A., Katzer, F., & Bartley, P. M. (2024). An abortion storm in a goat farm in the Northeast Region of Brazil was caused by the atypical Toxoplasma gondii genotype #13. *Current Research in Parasitology & Vector-Borne Diseases*, *5*, 100157. https://doi.org/10.1016/j.crpvbd.2023.100157

Villa, R., Perea, M., Aparicio, E. D., Mobarak, A. S., Andrade, L. H., & Gümes, F. S. (2008). Abortions and stillbirths in goats immunized against brucellosis using RB51, rfbK and Rev 1 vaccines. *Tecnica Pecuaria En Mexico*, *46*(3), 249–258.

Vogel, H., Daniels, J. B., & Frank, C. B. (2024). Nocardia farcinica abortion in a goat. *Journal of Veterinary Diagnostic Investigation : Official Publication of the American Association of Veterinary Laboratory Diagnosticians, Inc*, *36*(1), 128–130. https://doi.org/10.1177/10406387231210499

Vogt Engeland, I., Andresen, O., Ropstad, E., Kindahl, H., Waldeland, H., Daskin, A., & Olav Eik, L. (1998). Effect of fungal alkaloids on the development of pregnancy and endocrine foetal-placental function in the goat. *Animal Reproduction Science*, *52*(4), 289–302. https://doi.org/10.1016/s0378-4320(98)00107-9

Wagner, H., Eskens, U., Nesseler, A., Riße, K., Kaim, U., Volmer, R., Hamann, H.-P., Sauerwald, C., & Wehrend, A. (2014). Pathologic-anatomical changes in newborn goats caused by an intrauterine Schmallenberg virus infection. *Berliner Und Munchener Tierarztliche Wochenschrift*, *127*(3-4), 115–119. https://doi.org/10.2376/0005-9366-127-115

Waldeland, H., & Løken, T. (1991). Reproductive failure in goats in Norway: An investigation in 24 herds. *Acta Veterinaria Scandinavica*, *32*(4), 535–541. https://doi.org/10.1186/BF03546955

Wang, F. I., Shieh, H., & Liao, Y. K. (2001). Prevalence of Chlamydophila abortus infection in domesticated ruminants in Taiwan. *The Journal of Veterinary Medical Science*, *63*(11), 1215–1220. https://doi.org/10.1292/jvms.63.1215

Williams, N. M., Vickers, M. L., Tramontin, R. R., Petrites-Murphy, M. B., & Allen, G. P. (1997). Multiple abortions associated with caprine herpesvirus infection in a goat herd. *Journal of the American Veterinary Medical Association*, *211*(1), 89–91. https://doi.org/10.2460/javma.1997.211.01.89

Yong, H.-Y., Kim, S.-D., & Bae, B.-S. (2010). Termination of unwanted pregnancy in himalayan tahrs (Hemitragus jemlahicus) and a saanen goat (Capra hircus). *Journal of Veterinary Clinics*, *27*(2), 205–208. https://www.scopus.com/inward/record.uri?eid=2-s2.0-78049371792&partnerID=40&md5=16e127b167b2a789abeb00733ea5cfc7

Zainal Ulum, M. T., Ab Azid, Muhamad Affan, Shen, T. W., Hassim, H. A., Zamri-Saad, M., & Salleh, A. (2021). Histopathological changes in the reproductive organs of does with pregnancy toxaemia and their aborted foetuses. *Small Ruminant Research : The Journal of the International Goat Association*, *199 SP* -. https://doi.org/10.1016/j.smallrumres.2021.106363

Zaitsev, S., Khizhnyakova, M., Saltykov, Y., Evstifeev, V., Khusainov, F., Ivanova, S., Morozova, D., Yakovlev, S., Larionova, O., & Feodorova, V. (2024). Complete genome sequence of Chlamydia psittaci AMK-16, isolated from a small ruminant in the Middle Volga Region, Russia. *Microbiology Resource Announcements*, *13*(5), e0054323. https://doi.org/10.1128/mra.00543-23

Zundel, E., Verger, J. M., Grayon, M., & Michel, R. (1992). Conjunctival vaccination of pregnant ewes and goats with Brucella melitensis Rev 1 vaccine: Safety and serological responses. *Annales De Recherches Veterinaires*, *23*(2), 177–188. https://www.scopus.com/inward/record.uri?eid=2-s2.0-0026548693&partnerID=40&md5=866d6870682057c3139bb4b39611f5af
